# The spectrum of clinical biomarkers in severe malaria and new avenues for exploration

**DOI:** 10.1080/21505594.2022.2056966

**Published:** 2022-08-29

**Authors:** Loick Pradel Kojom Foko, Geetika Narang, Suman Tamang, Joseph Hawadak, Jahnvi Jakhan, Amit Sharma, Vineeta Singh

**Affiliations:** a Parasite and Host Biology Group, ICMR-National Institute of Malaria Research, New Delhi, India; b Molecular Medicine Group, International Centre for Genetic Engineering and Biotechnology, New Delhi, India

**Keywords:** Severe malaria, diagnostic, therapeutic, prognostic biomarkers, clinical infections

## Abstract

Globally, malaria is a public health concern, with severe malaria (SM) contributing a major share of the disease burden in malaria endemic countries. In this context, identification and validation of SM biomarkers are essential in clinical practice. Some biomarkers (C-reactive protein, angiopoietin 2, angiopoietin-2/1 ratio, platelet count, histidine-rich protein 2) have yielded interesting results in the prognosis of *Plasmodium falciparum* severe malaria, but for severe *P. vivax* and *P. knowlesi* malaria, similar evidence is missing. The validation of these biomarkers is hindered by several factors such as low sample size, paucity of evidence-evaluating studies, suboptimal values of sensitivity/specificity, poor clinical practicality of measurement methods, mixed *Plasmodium* infections, and good clinical value of the biomarkers for concurrent infections (pneumonia and current COVID-19 pandemic). Most of these biomarkers are non-specific to pathogens as they are related to host response and hence should be regarded as prognostic/predictive biomarkers that complement but do not replace pathogen biomarkers for clinical evaluation of SM patients. This review highlights the importance of research on diagnostic/predictive/therapeutic biomarkers, neglected malaria species, and clinical practicality of measurement methods in future studies. Finally, the importance of omics technologies for faster identification/validation of SM biomarkers is also included.

## Introduction

Tremendous efforts are being made worldwide to fight against malaria, but the disease remains an important public health concern, with 241 million cases and 627,000 deaths estimated in 2020 [1]. Malaria is caused by a *Plasmodium* protozoan parasite transmitted to humans through the infectious bite of female Anopheline mosquitoes during a blood meal. Five *Plasmodium* species can elicit human malaria *viz*. *P. falciparum* (*Pf*), *P. vivax* (*Pv*), *P. malariae* (*Pm*), *P. ovale* spp (*Po*), and *P. knowlesi* (*Pk*), with *Pf* and *Pv* responsible for the major bulk of malaria burden worldwide [1,2].

The clinical spectrum of malaria is diverse, going from asymptomatic carriage of parasites to clinical malaria with varying degrees of signs/symptoms that define non-severe and severe malaria [3]. A large number of factors (e.g. host genetics, immune status, drug resistance phenotype, host behavior, and concurrent infections) in the complex parasite—host—environment interaction modulate the clinical symptomatology in severe malarious patients [3]. Severe malaria (SM) is more frequently seen in patients infected with *Pf* and *Pv* [3], but recent reports have shown the ability of *Pm*, *Po,* and *Pk* to also elicit SM [4–6].

In clinical practice, a good management of SM is achievable in endemic areas. However, SM management is very tricky in some areas, especially those from sub-Saharan Africa (sSA) that have a proportionally high share (>90%) of world malaria morbidity and mortality [1], with a large number of remote areas and hard-to-reach populations. Skilled care providers, efficient good quality diagnostic tools, and antimalarial drugs are often missing in these areas, and thus, the management of SM is very challenging in such areas. In Sudan and Zimbabwe – two sSA countries – it was seen that the management of SM was suboptimal in the hospitals especially due to shortage of i) care providers, ii) availability of supplies (diagnostic test and treatment), and iii) the quality of care provided [7,8].

SM mainly due to *Pf* accounts for ~500,000 deaths annually, where children aged below five years old, pregnant women, and nonimmune travelers are major victims [9]. However, there is paucity of data on the real extent of SM in endemic areas mostly [10]. Using a systematic review and meta-analysis, we have shown that the overall proportion of *Pv* mono-infection-related SM is 22.9% in India [11]. A recent modeling study showed the cost-effectiveness of implementing interventions aiming at reducing SM in conjunction with standard measures for reducing costs and health burden due to malaria [12].

In the present review, we evaluated clinical biomarkers for their potential in the diagnosis, prognosis, prediction, and therapy of SM, identified the potential gaps, and proposed areas for future investigations/solutions, with a focus on omics technologies and their possible utility in the current research era.

## Severe malaria

**Figure 1.**
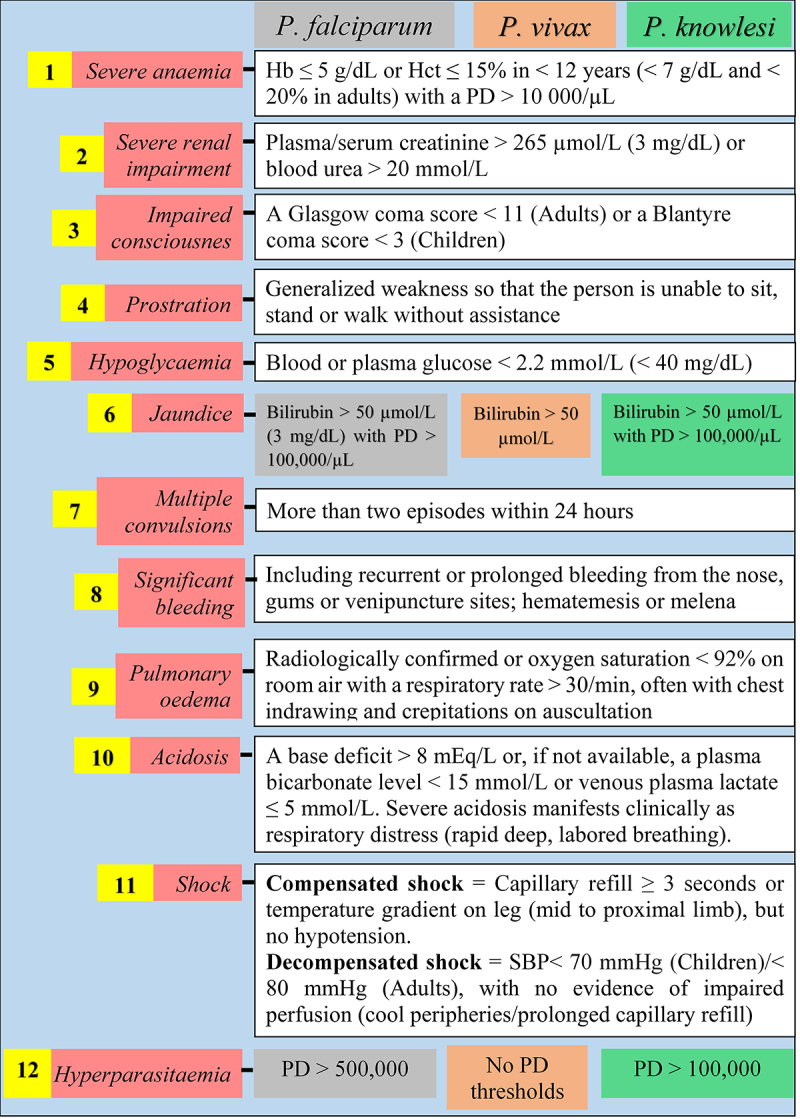
Latest WHO guidelines on the clinical presentation of severe malaria [18].

Since the 1990s, the World Health Organization (WHO) defined guidelines to diagnose SM based on findings from field and clinical studies. These guidelines have been regularly revised from 2000 to 2015 [13–18]. For SM diagnosis, the clinical symptoms mainly include severe malarial anemia (SMA), cerebral malaria (CM), hypoglycemia, multiple convulsions, prostration, acute respiratory distress syndrome (ARDS), pulmonary edema, acute renal failure, multiorgan dysfunction, jaundice, shock, bleeding, and hyperparasitemia (Figures 1 and Figure 2A). In malarious patients, several of these symptoms can exist together, leading to death within hours or days [19], and can be strong predictors in SM patients' recovery/survival [20]. Few studies that addressed the risk factors of SM listed some frequently identified factors as distance to the nearest health facility, concurrent comorbidities, nonuse of preventive methods, immune status, pregnancy, duration of illness before receiving antimalarial drug, delayed care, seeking self-medication, and patients' age [21–28]. For instance, Xia and colleagues have recently found that the risk of SM was higher in females and patients' aged ≥50 years old in the Hubei Province, a low malaria endemic area in China [28]. Few unusually addressed risk factors such as ethnicity have also been identified among *Pf*-infected adults [29].

Hb: hemoglobin, Hct: hematocrit, PD: parasite density, SBP: systolic blood pressure.

*These signs/symptoms are variably defined with respect to the *Plasmodium* species.

Definitions of *Po* and *Pm* severe malaria are not stated in the guidelines.

**Figure 2A.**
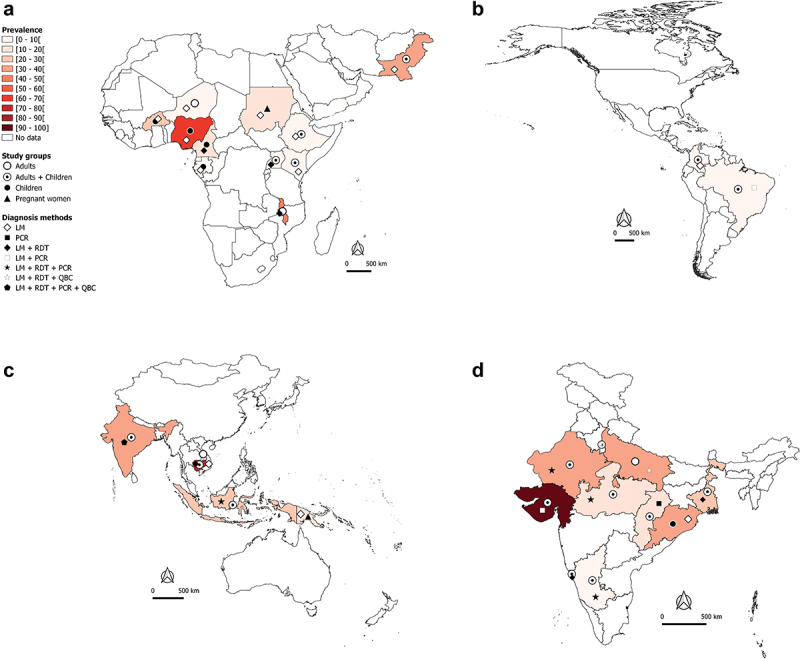
Worldwide burden of severe malaria.

Figure 2A(1-4) In-hospital-attended patients due to *Plasmodium* spp species.

Figure 2B(1-4) *P. falciparum* mono-infections.

Figure 2C(1-4) *P. vivax* mono-infections in African and Eastern Mediterranean regions (A1, B1, C1), The Americas region (A2, B2, C2), South East Asia and West Pacific regions (A3, B3, C3), and India (A4, B4, C4), 2010—2020.

In Figure 2A(1-4), the total number of SM cases irrespective of the malaria species identified in studies was used to determine the proportion of SM in a country or an area.

Weighted proportions were calculated to depict hospital-admitted SM. For a same country or area, the weight of a study was determined by dividing the sample size of admitted patients in this study by the total number of admitted patients of all studies conducted in the country or area. The overall estimate of the SM proportion was then computed by summing up the product of SM prevalence in each study and the weight of the study.

LDR-FMA: ligase detection reaction-fluorescent microsphere assay, LM: light microscopy, PCR: polymerase chain reaction, QBC: quantitative buffy coat, RDT: rapid diagnostic test.

The maps were generated using the QGIS software.

The data are sourced from research articles retrieved from PubMed (https://pubmed.ncbi.nlm.nih.gov/), published in English and French languages We excluded studies with a small sample size, case—control design, published before 2010, imported malaria, studies with no country-wise data, and reviews/abstracts/book chapters.

**Figure 2B.**
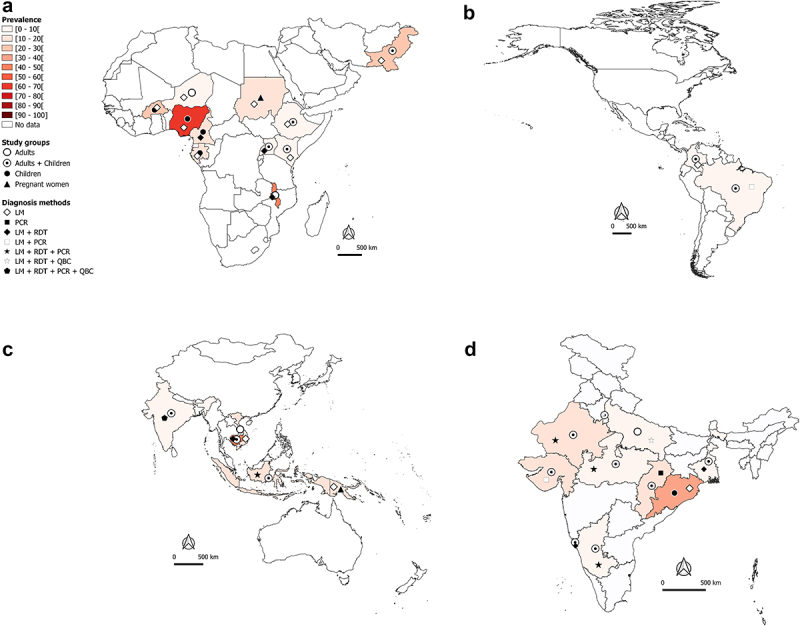
Continued.

**Figure 2C.**
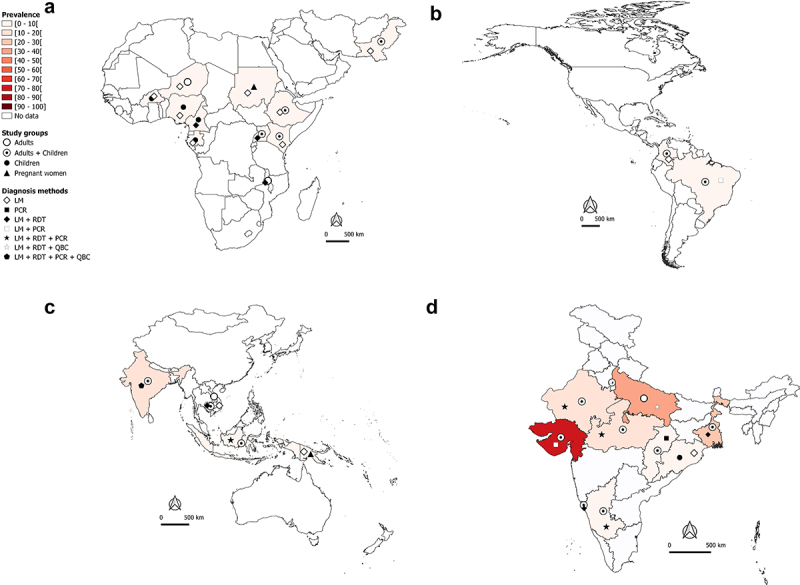
Continued.

## Worldwide burden of severe malaria in hospital-admitted patients

In the African and Eastern Mediterranean regions, SM may account for 40–50% of hospital-admitted malaria cases in countries such as Burkina Faso, Malawi, and Pakistan (Figure 2A). In Nigeria and Cambodia, the prevalence of hospital admitted SM due to *Plasmodium* regardless of species is very high (>70%) in clinical malaria cases with majority being *Pf* cases (Figure 2A). Data available and presented in Figure 2B suggest that *Pf* is mainly responsible for hospital admitted SM cases in sSA regions. In the Americas, the SM prevalence is relatively low (<10%), with *Pf* and *Pv* both involved in hospital admitted SM cases, but with a clear predominance of *Pv* in Brazil and Colombia (Figure 2C). In the South East Asia (SEA) region, the bulk of SM data comes from India. The extent of involvement of *Pf* and *Pv* in SM cases varies between the different states of the country. In Orissa, *Pf*-SM burden is the highest (40–50% of the malaria cases) as compared to other states (Figure 2B), whereas *Pv* species is predominantly responsible for hospital admitted SM cases in several states, including Delhi, Gujarat, Maharashtra, Madhya Pradesh, Uttar Pradesh, Rajasthan, and West Bengal (Figure 2C).

## The need for clinical biomarkers of severe malaria

A biomarker is a characteristic presentation (e.g. clinical, genetic, and hematological) or a substrate (e.g. proteins and metabolites) that can objectively be measured and evaluated as an indicator of normal biological/pathogenic processes or response to a therapeutic intervention [30]. In malariology, it can be particularly helpful in clinical practice and public health from disease diagnosis to identification of patients at risk for long-term complications (Figure 3A).

**Figure 3A.**
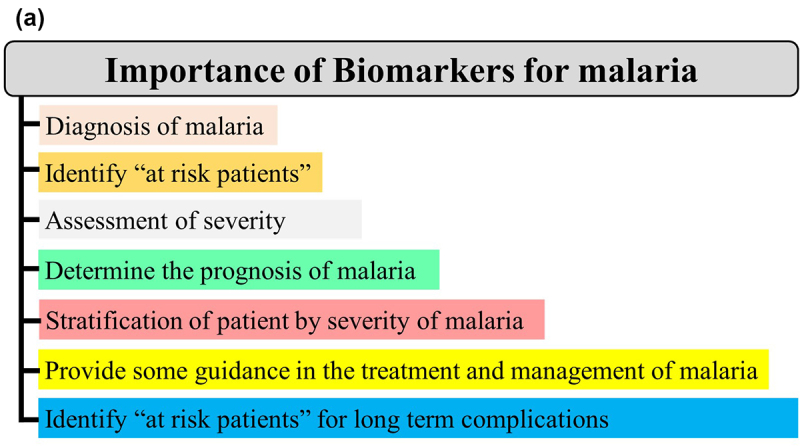
Continued.

Biomarkers can be classified into four types *viz* diagnostic, prognostic, predictive, and therapeutic. Generally, a **diagnostic biomarker** allows the early detection of a disease in a noninvasive way, leading to its prevention. A **prognostic biomarker** is a clinical or biological characteristic that is objectively measurable and that provides information on the likely outcome of the disease in an untreated individual [31–33]. A **predictive biomarker** is a clinical or biological characteristic that reveals information, allowing one to forecast the response of patients to a given treatment and thus identify individuals who will benefit from the treatment [31–33]. A **therapeutic biomarker** is most commonly a protein that can be used as a target for disease therapy (Figure 3B).

**Figure 3B.**
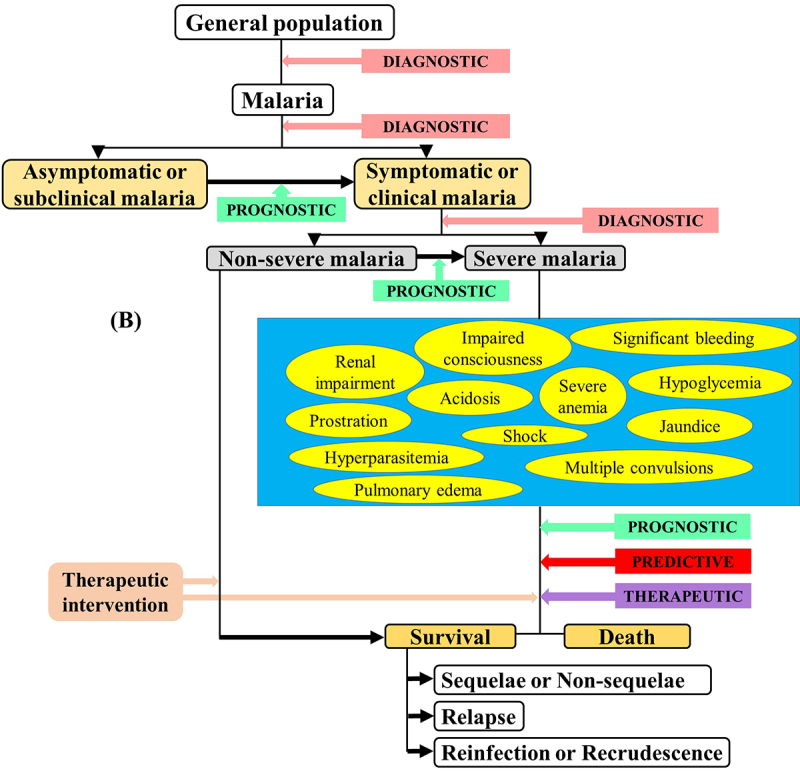
Importance (A) and categorization (B) of biomarkers in malaria infection.

In clinical practice, prognostic markers can allow one i) to discriminate untreated asymptomatic malaria patients susceptible to manifest clinical signs/symptoms, ii) identify untreated clinical patients whose malaria infection could lead to a severe form, and iii) identify untreated SM patients (Figure 3B). Thus, the identification of such markers for SM is of utmost importance as they will facilitate in the reduction of SM burden.

## Evaluation and validation of clinical biomarkers

The characteristics of an ideal biomarker have been described previously with the following attributes: i) clinical relevance, ii) good sensitivity, specificity, and predictive values, iii) reliability, iv) simplicity, and v) practicality [33,34].

The clinical relevance of a biomarker refers to the evidence that supports a coherent basis of its utility (e.g. the ability of a biomarker to reflect any aspect of the pathological process, in this context, SM). Sensitivity refers to the ability of a biomarker to correctly identify patients with the disease-related outcome (e.g. mortality risk and severity outcome), while specificity refers to the capacity of a biomarker to correctly identify patients exempted from the disease-related outcome. The determination of the area under curve (AUC) of the biomarker by plotting a receiver operating characteristic curve (ROC curve) can also be used as an alternative to sensitivity and specificity. In practice, a biomarker with sensitivity (Se) ≥85%/specificity (Sp) ≥75% or an AUC ≥0.75 is considered to be of good clinical utility [35–37]. A reliable biomarker that can be quantifiable should have acceptable precision, accuracy, reproducibility, and robustness. The quantitative evaluation of the biomarker should be simple to perform (i.e. without the need for a skilled operator and costly equipment) and as least invasive as possible in order to obtain approval of patients and apparently healthy individuals. According to the nature of the biological sample used for quantifying the biomarker, The Ronald and Nancy Reagan Research Institute of the Alzheimer’s Association and the National Institute on Aging Working Group have categorized the invasive methods into three categories such as noninvasive (blood, urine, saliva, or buccal scrapings), moderately invasive (skin or rectal biopsies, cerebrospinal fluid-CSF, or bone marrow), and highly invasive (brain tissue) [35]. These different criteria are used to evaluate the performances and clinical utility of biomarkers.

The validation of biomarkers is the guarantee underlying the subsequent production of high-quality research data on diagnosis, prognosis, and prediction of the SM outcome. The procedure for the validation of biomarkers is multifaceted and more complex than their evaluation where validation takes into account the above-mentioned criteria on evaluation along with additional aspects such as the study design (sample size, characteristics of cases/controls), reproducibility in varied settings, pharmacokinetics—pharmacodynamics data, establishment of the biomarker normal range, and biological plausibility [33,34].

## Biomarkers for severe malaria human *Plasmodium* species

The literature analysis of the related topic showed very few studies that evaluated the prognostic potential of few biomarkers for SM. One study addressed the evaluation of the platelet count and plateletcrit as SM prognostic markers without any distinction of the involved malaria species [38]. A cross-sectional study conducted on Indians aged ≥18 years evaluated the prognostic value of the platelet (PLT) count and plateletcrit to discriminate SM patients from non-SM patients. The authors found that a PLT of 50,000/µL had a sensitivity of 65.6%, a specificity of 70.6%, and an AUC of 0.713. The values of sensitivity and specificity were similar for a plateletcrit of 0.05% but had an AUC of 0.718 [38].

## Biomarkers for severe *Pf* malaria

**Figure 4.**
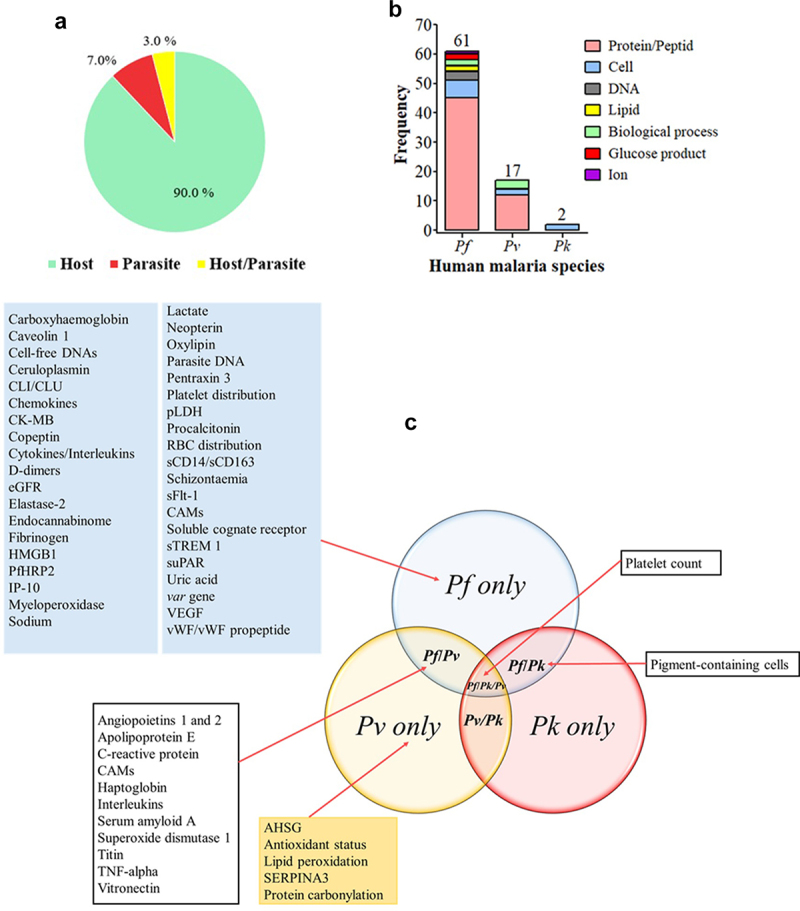
Clinical biomarkers and severe malaria.

**Figure 5.**
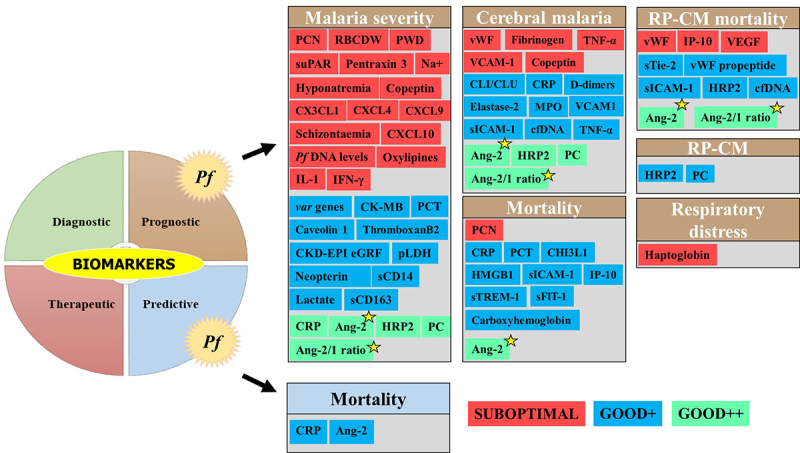
Biomarkers evaluated for severe *Pf*-SM.

A large number of biomarker-related aspects have been extensively studied for this malarial species for the identification of i) patients at SM risk, ii) markers of SM outcomes, iii) patients at risk of CM, SMA, shock, ARDS/pulmonary edema, and renal failure, iv) patients with chances to recover from an SM episode, and v) CM patients at fatal risk or survival with the development of neurological sequelae (Supplemental material 1 and Figure 5). Most of the *Pf* biomarkers evaluated were host proteins (Figure 4a–b).

Figure 4A Origin of biomarkers.

Figure 4B Biological nature of biomarkers by malaria species.

Figure 4C Type of biomarkers evaluated.

Biomarkers for which reporting AUC/sensitivity/specificity values were included.

In 4B, the numbers above the histograms represent the total number of evaluated biomarkers for each malaria species.

In 4C, some biomarkers were studied in more than one malaria species (e.g. the platelet count was evaluated as biomarkers of severe malaria with *Pf, Pv,* or *Pk*).

AHSG: alpha-2-Heremans-Schmid glycoprotein, AUC: area under the curve, CAMs: cell adhesion molecules, CK-MB: cardiac disease creatine kinase muscle-brain type, CLI/CLU: circulatory complement‑lysis inhibitor or clusterin, DNA: deoxyribonucleic acid, eGRF: estimated glomerular filtration rate, HMGB1: high-mobility group box protein 1, HRP2: histidine-rich protein 2, IP-10: 10-kDa INF gamma-induced protein, *Pf*: *Plasmodium falciparum*, *Pk*: *Plasmodium knowlesi*, *Pv*: *Plasmodium vivax*, pLDH: parasite lactate dehydrogenase, RBC: red blood cell, sICAM-1: soluble intercellular cell adhesion molecule 1, SERPINA3: serpin peptidase inhibitor clade A member 3, sTie-2: soluble cognate receptor, sTREM 1: soluble triggering receptor expressed on myeloid cells 1, suPAR: soluble urokinase-type plasminogen activator, TNF: tumour necrosis factor, VEGF: vascular endothelial growth factor, vWF: von Willebrand factor.

Biomarkers for AUC/sensitivity/specificity values were included.

Biomarkers for severe *Pf* malaria were categorized into three groups: i) suboptimal [the biomarker has AUC and/or sensitivity and specificity values below thresholds], ii) Good+ [the biomarker has AUC and/or sensitivity and specificity values above thresholds, but with limited evidence of its clinical utility due to several reasons, including a low number of studies (n = 1), no statistical significance was provided for AUC, evaluated in a specific population (i.e. nonimmune European travelers and evaluated by the same research team], and iii) Good++ [the biomarker has AUC and/or sensitivity and specificity values above thresholds, and strong evidence of its clinical utility due to several reasons including number of studies > 1, statistical significance provided for AUC, evaluated in different malaria endemic settings, evaluated in populations from malaria endemic areas, and evaluated by different research teams]. The Ang-2 and Ang-2/1 ratio (yellow star) showed good prognosis performances for most of the SM-related outcomes.

Ang: angiopoietin, CCL: chemokine ligand, cfDNA: cell-free deoxyribonucleic acid, CHI3L1: chitinase-3-like 1 protein, CRP: C-reactive protein, DNA: deoxyribonucleic acid, HMGB1: high-mobility group box protein 1, HRP2: histidine-rich protein 2, IFN: interferon, IL: interleukins, IP-10: 10-kDa INF gamma-induced protein, PC: platelet count, PCN: pigment-containing cells, PCT: procalcitonin, *Pf*: *Plasmodium falciparum*, PWD: platelet width distribution, RBCDW: red blood cell distribution width, RP: retinopathy positive, sICAM-1: soluble intercellular cell adhesion molecule 1, sTie-2: soluble cognate receptor, suPAR: soluble urokinase-type plasminogen activator, TNF: tumor necrosis factor, VCAM-1: vascular adhesion molecule 1, VEGF: vascular endothelial growth factor, vWF: von Willebrand factor.

Among the *Pf*-SM biomarkers, five prognostic biomarkers *viz* CRP, Ang-2, Ang-2/1 ratio, PfHRP2, and platelet count showed good clinical values in malaria severity and CM. For example, CRP and platelet count exhibited interesting performances of malaria severity in nonimmune European patients as well as in individuals from malaria endemic areas (India, Sudan, Nigeria, Senegal, and Malawi) (Supplemental material 1 & Figure 5) [39–44]. As reported for malaria severity and CM, the Ang-2 and Ang-2/1 ratio showed a good clinical value for the prognosis of mortality among SM cases and mortality among CM patients diagnosed with retinopathy [45–47] (Supplementary file 1 and Figure 5). On analysis of these five prognostic biomarkers, PfHRP2 seems to be the most discriminant for CM, while Ang-2 and Ang-2/Ang-1 seem to be the best indicator for malaria severity. In addition, the Ang-2 and Ang-2/1 ratios seem to be the most promising prognostic biomarkers as they showed good performances for most of the SM-related outcomes (i.e. malaria severity, CM, mortality, and RP-CP mortality) (Figure 5).

Other biomarkers that were included in the list have shown preliminary good performances, but the evidence of their clinical utility is still limited by the low number of studies, evaluation of specific populations only (i.e. nonimmune travelers), and the absence of statistical significance. Such biomarkers are procalcitonin (PCT), *var* genes, haptoglobin, neopterin, circulatory complement‑lysis inhibitor or clusterin (CLI/CLU), cardiac disease creatine kinase muscle-brain type (CK-MB), uric acid, chronic kidney disease-epidemiology estimated glomerular filtration rate (CKD-EPI eGFR), total bilirubin, caveolin 1, parasite lactate dehydrogenase (pLDH), high mobility group box protein 1 (HMGB1), oxylipin and endocannabidome metabolites, D-dimers, cell-free DNA (cfDNA), and carboxyhemoglobin (Supplemental material 1 and Figure 5). The prognostic value of CM biomarkers was also greatly influenced by the host immunity as good performances of cell adhesion molecules (VCAM-1, sICAM-1) were reported in European travelers, but in contrast, poor performances were found in Ugandan children [48,49].

Finally, biomarkers such as, for instance, pigment-contained neutrophils (PCNs), fibrinogen, copeptin, some chemokines (CX3CL1, CXCL4, CXCL9, and CXCL10), IP-10, and schizontaemia showed poor clinical performances for their ability to discriminate the above-mentioned SM outcomes (Figure 5).

## Biomarkers for severe *Pv* malaria

**Figure 6.**
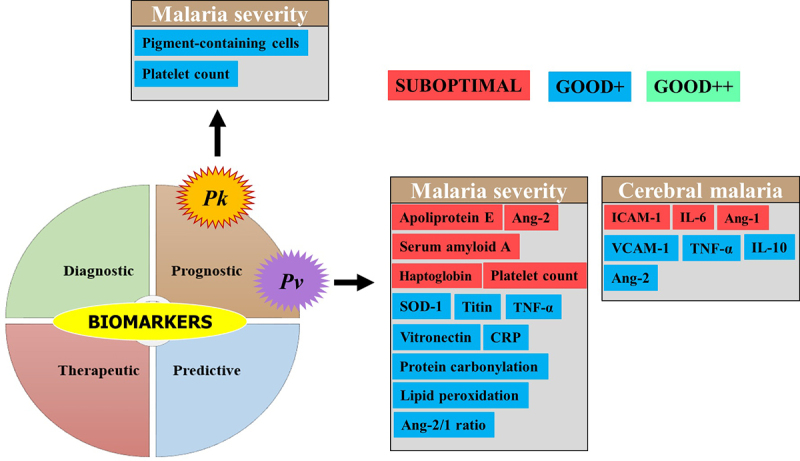
Biomarkers evaluated for severe *Pv* and *Pk* malaria.

Several studies addressed biomarkers in *Pv*-SM, although the number of biomarkers evaluated is far lower than that seen in *Pf*-SM. To date, no strong evidence of the clinical utility of biomarkers is available for *Pv*-SM even though some of them such as superoxide dismutase 1 (SOD), titin, vitronectin, TNF-α, and the Ang-2/1 ratio have shown interesting performances for the prognosis of malaria severity (Figure 6). Other CM biomarkers (IL-10, VCAM-1, TNF-α, and Ang-2) for potential prognosis were seen in studies from Brazil, India, and Pakistan [50–52]. In contrast, poor performances, especially for malaria severity prognosis, were reported for other biomarkers, including IL-6, haptoglobin, apolipoprotein E, serum amyloid A, Ang-1, ICAM-1, and platelet count (Supplemental material 2 and Figure 6).

Biomarkers for AUC/sensitivity/specificity values were included.

Biomarkers for severe *Pv* and *Pk* malaria were categorized into three groups: i) suboptimal [the biomarker has AUC and/or sensitivity and specificity values below thresholds], ii) Good+ [the biomarker has AUC and/or sensitivity and specificity values above thresholds, but with limited evidence of its clinical utility due to several reasons including a low number of studies (n = 1), no statistical significance was provided for AUC, evaluated in a specific population (i.e. nonimmune European travelers, and evaluated by the same research team], iii) Good++ [the biomarker has AUC and/or sensitivity and specificity values above thresholds, and strong evidence of its clinical utility due to several reasons including number of studies > 1, statistical significance provided for AUC, evaluated in different malaria endemic settings, evaluated in populations from malaria endemic areas, and evaluated by different research teams].

Ang: angiopoietin, CRP: C-reactive protein, IFN: interferon, IL: Interleukin, *Pk*: *Plasmodium knowlesi*, *Pv*: *Plasmodium vivax*, sICAM-1: soluble intercellular cell adhesion molecule 1, SOD-1: superoxide dismutase 1, TNF-α: tumor necrosis factor alpha, VCAM-1: vascular adhesion molecule 1.

## Biomarkers for severe *Pk* malaria

*Plasmodium knowlesi* is mostly found in SEA especially in Malaysia where its prevalence and burden often outdo those of *Pf* species [5]. Platelet count and PCN were evaluated for their prognostic value toward SM in Malaysian adults and were found to show similar performances between the platelet count (AUC = 0.77) and PCN (AUC = 0.85) compared to the parasite density (AUC >0.80) (Supplemental material 2) [53]. Another study found that plasma Ang-2 and osteoprotegerin levels were significantly higher in the plasma levels of Malaysian SM patients aged below 18 years old, and these two proteins independently were risk factors for renal impairment, despite the fact that the authors did not evaluate their malaria severity-related sensitivity and specificity [54]. Despite these encouraging results, evidence of their clinical utility for *Pk*-SM is still insufficient (Figure 6).

## Biomarkers proposed for severe *Pm* and P*o* malaria

There are no studies for the evaluation of SM biomarkers for these two species, and this is likely due to a very low number of *Po*- and *Pm*-related morbidity and mortality cases, worldwide [4,6]. Based on a systematic review and meta-analysis, our research group has recently shown that the global prevalence of these two species was 2.01% for *Pm* and 0.77% for *Po* spp, with the highest values recorded in sSA (3.16% for *Pm* and 1.69% for *Po*) [55].

## SM biomarkers' critical evaluation, challenges, and future research

The evaluation and validation of reliable biomarkers for SM are crucial to develop efficient strategies to manage this important public concern worldwide. In the present review, it is obvious that this research era is still in its infancy, given the paucity of data on potential biomarkers. The studies were mainly focused on the prognostic aspect of the evaluated biomarkers rather than on their predictive and therapeutic aspects. The forecasting of the probable occurrence of SM is important, but the identification of patients with less chances of recovery from an antimalarial therapy (i.e. predictive biomarkers) and identification of biomolecules susceptible to be targeted by this therapy (i.e. therapeutic biomarkers) are also important aspects of management of SM by practitioners. Some studies outlined an association of elevated levels of biomarkers such as Ang-1, sTREM-1, CXCL10, and sICAM-1, with prolonged clinical recovery times in patients having survived from *Pf*-SM disease [56], while other putative biomarkers were not found to be associated with *Pf*-SM-related clinical recovery and time [57]. Despite the fact that such findings were reported in some studies, it would be premature to conclude a possible use of these biomolecules as potential predictive and/or therapeutic biomarkers as the studies were not properly designed to address these aspects. We noted that some studies concluded on a possible value of some biomarkers based on the statistical evidence of a difference in body fluid levels or levels of genomic expression between their different groups (e.g. healthy control – HC, uncomplicated malaria – UM, mild malaria – MM, severe malaria – SM, and cerebral malaria – CM) or an OR-based quantification of SM risk or mortality and have not given any estimates of sensitivity, specificity, predictive values, and AUC. These parameters should be given impetus by research community and practitioners for effective clinical impact of the putative biomarkers.

Some biomarkers have been found to produce relatively good and statistically significant prognostic performances in terms of AUC, sensitivity, and specificity, which include i) CRP, circulatory complement‑lysis inhibitor, Ang-2, HRP2, thromboxane 2, cfDNA, platelet count, TNF-α, and 10-kDa INF gamma-induced protein for *Pf* and ii) SOD-1, TNF-α, protein carbonylation, lipid peroxidation, IL-10, and cell adhesion molecules for *Pv* (Supplementary files 1 2, Figure 5). Unfortunately, the findings presented come either from only one study or from several studies of the same research group or from the same geographical setting, thereby limiting the evidence. In addition, the design of studies was not comparable and some studies addressed different aspects of SM (i.e. malaria severity, CM only, mortality, etc.). The demonstration of the usefulness of a given biomarker requires the implementation of more evidence-providing studies in different contexts and by different research teams. Case—control designed studies are better than retrospective and cross-sectional studies to evaluate any potential clinical biomarker. The evaluation of the behavior of biomarkers in other malaria vulnerable groups such as pregnant women and immunocompromised people other than children is also encouraged as they are majorly affected by malaria disease, especially SM.

On studying individually, we noted that a large proportion of biomarkers did not qualify as prognostic markers for SM, as no evidence of statistical significance or performance criteria values below the acceptable thresholds was available. One way to improve the potential of biomarkers could be to use them in combination as seen in previous studies [45,58–63]. Many reasons can explain poor individual performances of these biomarkers:

- small sample size of cases and controls impacting greatly on the data analysis, statistical test findings, and finally the interpretation of data. Thus, the implementation of adequately powered studies is a prerequisite to support the findings;
- findings on the accuracy of methods used to determine the levels of biomarkers in biological fluids are rarely given in the studies and absence of taking into account this aspect may give prejudices to results;
- the presence of latent concurrent infections such as viral, parasitic, and bacterial infections or conditions like malnutrition, which also may modulate the levels of biomarkers investigated and severity of malaria disease. Few studies used concurrent infections such as dengue to identify and evaluate the potential of some biomarkers for severe *Pv* malaria [64,65]. Other studies showed a good prognostic value of Hp and Lpc-2 to distinguish severe pneumonia due to malaria from those of bacterial and viral origin [58,66], while few studies found CRP to be a good biomarker in the current coronavirus pandemic (COVID-19) [67,68].

The validation of SM biomarkers should also overcome the problem of mixed infections with plasmodial species. In some settings, the proportion of co-infections is often surprisingly high as reported for *Pf-Pm* co-infection from North-Western part of Cameroon [69]. The proportion of mixed *Plasmodium* infections is also increasing in sSA as reported recently in a meta-analysis where 9% of mixed infections led to SM and that the proportion of pulmonary complications and severe anemia were higher in patients with mixed infections [70]. Even though low proportions of mixed infections are reported from malaria endemic settings, whether these mixed infections influence the biological dynamics and usefulness of biomarkers remains an elusive question still.

The PfHRP2 protein has shown good performances for CM prognosis and distinction between CM malaria patients with positive and negative diagnosis for retinopathy (CM-RP and CM-RN) (Supplementary file 1 and Figure 5). The recent reports on the appearance of *Pf* parasites with *pfhrp2* deletions could be an important concern on the long-term utilization of PfHRP2 as a diagnostic biomarker for *Pf* infections where it is highly predominant in settings as in sSA and some areas of India [71–73] and could also be an obstacle to its utilizationin the prognosis of CM and distinction between CM-RP and CM-RN cohorts.

The *Pk* species is majorly present in SEA, especially in Malaysia where its prevalence is very high in some areas of the country [74–76]. For severe *Pk* malaria, no promising biomarkers have been reported till now. Some putative biomarkers of *Pk* malaria infection such as Hpx, haptoglobin, and serotransferrin were proposed [77], and it would be interesting to evaluate their potential in severe *Pk* malaria.

Even though a good biomarker needs to be validated for SM, the clinical practicality of the methods used to measure this biomarker is also a crucial parameter. ELISA is commonly used to measure the putative biomarkers along with other methods including molecular methods (i.e. real-time PCR, genotyping/cloning), flow cytometry, light microscopy, immunocolorimetric assays, co-oximetry, and indirect potentiometry (Supplemental materials 1 and 2). Routine implementation of these tests at clinical sites is greatly jeopardized by drawbacks such as high cost, time consumption, extensive training/expertise/workload for clinical staff, sensitivity/specificity, and reproducibility (Table 1). It would be interesting to address the cost-effectiveness of these different methods and develop point-of-care technologies in the context of biomarkers for SM, especially in resource-constrained and remote areas.

**Table 1.** Comparison of quantitative methods of SM biomarkers.

Techniques	Analysis time	Training	Cost	Workload	Reproducibility	Instrumentation required
Automated immunofluorescent assay^f^	Minutes to hours	Low	Moderate	High	Moderate to high	Colorimeter/microscope
Bradford assay^a^	Minutes	Low	Low	Low	Low to high	Spectrophotometer
Di-nitrophenyl hydrazine assay^a^	Minutes	Low	Low	Low	Low	Spectrophotometer
ELISA^a^	Hours	Low	Low to high	Low	High	Colorimeter/spectrophotometer
Flow cytometry^f^	Minutes	High	High	Low	High	Flow cytometer
FRAP assay	Minutes	Low	Low	Low	High	Spectrophotometer
Genotyping/cloning	Several hours to days	High	High	High	High	Thermocycler
Hematological analyzer	Minutes	Low	High	Low	High	Robot
Immunocolorimetric assay^a^	Minutes	Low	Moderate to high	Low	High	Spectrophotometer
Co-oximetry^f^	Minutes	Low	Moderate	Low	High	Oximeter
Indirect potentiometry^f^	Minutes	Low	Moderate	Low	High	Potential measuring device
Electrolyte analyzer	Minutes	Low	Moderate	Low	High	Robot
Light microscopy	Minutes to hours	High	Moderate	High	Low to high	Light microscope
Quantitative PCR	Minutes to hours	High	High	Low	High	Real-time thermocycler
Stable isotope dilution	Minutes to several days	High	Moderate to high	High	Low to high	Spectrophotometer
Thiobarbituric acid reaction assay^a^	Minutes to hours	Low	Moderate to high	Low	Moderate to high	Spectrophotometer
Turbidimetric immunoassay^ae^	Minutes	Low	Moderate to high	Low	High	Turbidimeter (turbidity meter)
B·R·A·H·M·S PCT-Q test^f^	30 min	Low	Low	Low	High	-

aThese are commercial kits.

bThis method is used to measure the platelet count.

cThis method is used to measure blood sodium.

dThis method is used to measure blood carboxyhemoglobin.

eThis method is used to measure C-reactive protein.

fThis is a one-step immunochromatographic assay-based point-of-care detecting Procalcitonin using immunogold labeling.

The few potentially helpful biomarkers identified here are mainly related to host response and they are therefore more likely suitable as prognostic and/or predictive biomarkers that complement but do not replace or substitute for diagnostics in the clinical evaluation and management of malaria patients. Biomarkers of host response are pathogen non-specific as mentioned in bacterial and COVID-19 infections and do not indicate the pathogen responsible for the clinical syndrome of “sepsis,” as represented by SM. As such, the biomarkers of host response provide insight into the potential risk for end-organ injury and adverse clinical outcomes. For example, biomarkers can potentially provide risk of progression to SM upon presentation of a patient with malaria (e.g. Ang-2).

**Figure 7.**
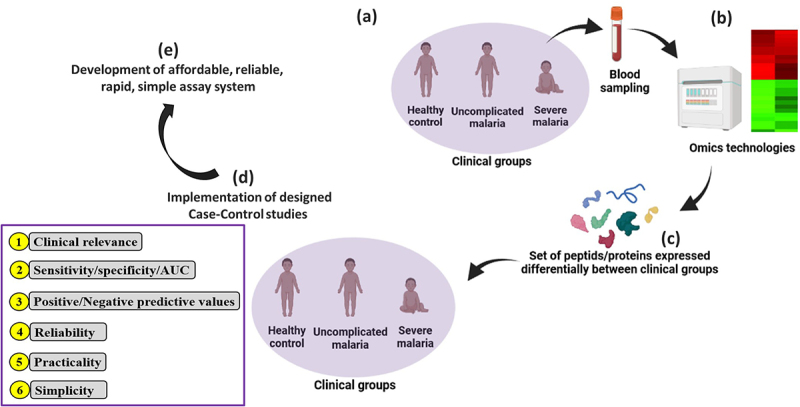
Utility of omics technologies to accelerate the identification and validation of clinical biomarkers for SM.

The investigation of new potentially promising candidate biomarkers is also crucial, and with the advent of transcriptomic, peptidomic, and proteomic technologies, the process of identification, evaluation, and validation of biomarkers for different aspects of malaria severity as seen in previous studies can be accelerated [64,65,78–80] (Figure 7). In Table 2, some candidate biomarkers such as apolipoprotein A-I, hemopexin (Hpx), apolipoprotein E, retinol-binding protein 4 (RBP4), ceruloplasmin, and plasminogen were identified using these technologies and further validated through estimates of their performances (i.e. sensitivity, specificity, predictive values, and AUC) using adequately designed studies. The cost, complexity, and time-consuming nature of omics technologies are major obstacles to their real-time clinical application in malaria settings, which are predominantly resource-limited areas. Another crucial challenge in the usage of such advanced technologies is to be able to translate “omics” technology-identified potential biomarkers into a relatively simple, rapid, reliable, low-cost assay (e.g. immunoassay) and make it commercially available in malaria endemic regions.

(a) Blood samples are collected from a small number of individuals based on symptomatology of malaria (i.e. no malaria/healthy control, uncomplicated malaria, and severe malaria). These different clinical groups are defined based on the objectives of the study. (b) Samples are processed using omics technologies to identify RNA/protein/peptide expression patterns between these different clinical groups in order to identify differentially expressed molecules. (c) The most differentially expressed biomolecules are identified and characterized (d) Case–control studies may be implemented to evaluate the clinical value of these sets of selected biomolecules. Parameters such as sensitivity, specificity, and area under the curve can be evaluated. Other aspects such as reliability, simplicity, and practicality of the measurement method can be evaluated in parallel. In this scenario, three clinical groups were used as an example, but design could be made more complex by defining or adding supplementary clinical groups (e.g. i—severe *Pf* + *Pv* cases, ii—different severe malaria forms as cerebral malaria, severe malarial anemia, respiratory distress, iii—severe co-infections with *Plasmodium* and other pathogens – virus, bacteria, etc.) and then performing omics technologies as presented in the figure, and (e) the development of a rapid, reliable, and affordable assay system should be the final step for the utilization of clinical biomarkers.

**Table 2.** Some proposed severe malaria biomarkers for which clinical performances were not evaluated.

Biomarkers	Description	Author’s findings	References
**Osteoprotegerin**	Member of the TNF receptor family, but it is secreted and acts like a cytokine and plays a crucial role in osteoporosis.	The plasma level of this biomolecule was significantly higher in *Pk*-SM patients.	[54]
**Cell-free Hb**	Hb is released into the bloodstream during some RBC rupture inducing conditions as those seen in severe *Pf* malaria.	Cell-free Hb was significantly higher in *Pk*-SM as compared to *Pk*-UM and HC. Cell-free Hb was significantly correlated with parasitemia, lactate, creatinine, Ang-2, ICAM-1, and E-selectin.	[54]
**Micro** **RNAs**	MicroRNAs (miRNAs) are a class of endogenous small noncoding RNAs present in several cells (cellular miRNAs) and body fluids (circulating miRNAs).	MicroRNA 4497 is significantly more expressed in *Pf*-SM compared to *Pf*-UM patients. Other miRNAs (23a, 24, 27a, 125b, 181b, 150, and 210) are variably expressed, depending on the form of SM (i.e. CM, renal failure, pulmonary edema, ARDS, and metabolic acidosis).	[81–84]
**Erythropoietin (Epo)**	Epo is a protein mainly synthesized in the kidney in response to hypoxia and is crucial for the proliferation and differentiation of erythroid cell lines.	High levels of Epo (>200 units/L) were associated with a reduced risk of neurological sequelae in children with *Pf*-CM (OR = 0.18, 95% CI 0.01-0.93).	[85]
**Galectin-9**	Gal-9 is presumed to be involved in several physiological and pathological processes by binding molecules such as glycoproteins and glycolipids of immune cells and pathogens.	Gal‑9 levels were also higher in *Pf*-SM compared to *Pf*-UM.	[86]
**Granzyme B (GrzB)**	Member of a family of serine proteases expressed in the granules of natural killers and cytotoxic T cells.	Plasma GrzB levels were also higher in *Pf*-SM compared to *Pf*-UM in Ghanaian children.	[87]
**Cell-specific microparticles (MP)**	Fragments of the plasma membrane shed by various cell types under physiological stress conditions.	Platelet, erythrocyte, endothelial, and leukocyte MP levels were elevated in patients with cerebral dysfunctions and returned to normal by discharge. In *Pf*-CM patients, platelet MP were the most abundant and their levels significantly correlated with coma depth and thrombocytopenia.	[88]
**Synapsin I**	Member of the synapsin family produced in synaptic vesicles and involved in exocytosis transport mechanisms in synapses.	Significant increase in synapsin I in *Pf*-CM compared to non-CM and HC both in granule cells and glomerular synaptic complex.	[89]
**Pantetheinase (PA)**	Ubiquitous enzyme that hydrolyzes D-pantetheine into vitamin B5 (pantothenate) and cysteamine in the Coenzyme A pathway.	Patients with *Pf*-CM had lower PA than those with *Pf*-UM. *Pf*-CM patients had a lower serum PA than others with *Pf*-SMA or HC.	[90]
**Extracellular vesicles (EVs)**	EVs are lipid bilayer-delimited particles (30 nm to 4 µm) that are naturally released from a cell, and thus EVs can theoretically be released from any body cell type. EVs were thought to play only a structural function, but recent studies outline their role as mediators of crucial biological processes for several parasites including *Plasmodium* parasites (e.g. parasite virulence, cell invasion).	Some studies outlined decreased levels of some EVs in *Pf*-SM patients, especially in *Pf*-CM cases.	[91]
**Hemopexin (Hpx)†**	Hemopexin (Hpx) is a plasma protein belonging to the inflammation acute phase groups and mainly produced by the liver.	Hpx had good prognosis potential for discrimination between *Pf*-SM and *Pf*-UM patient cohorts. On admission, the Hemin-to-Hpx ratio was significantly higher in i) SMA patients vs non-SMA (0.124 *vs* 0.016, *p* < 0.0001) and ii) RD vs non-RD patients (0.063 vs 0.020, *p* < 0.01) in another study.	[78,92,93]
**Hemin to hemopexin ratio**	Hemin is an iron-containing porphyrin with chlorine that can be formed from a heme group such as heme B found in Hb.	On admission, the ratio was significantly higher in i) *Pf*-SMA *vs Pf*-non-SMA (0.124 *vs* 0.016, *p* < 0.0001)and ii) *Pf*-RD *vs Pf*-non-RD patients (0.063 *vs* 0.020, *p* < 0.01). This ratio was not associated with 48-h mortality (short-term) but was associated with 6-month mortality (long-term) (*p* = 0.012).	[93]
**Ceruloplasmin†**	Ceruloplasmin is a copper-containing glycoprotein found in the α2 globulin fraction of human serum. It is involved in the pathological process of several iron- and copper-related metabolic disorders such as Wilson’s disease.	Its plasma level was altered depending on the severity of malaria. Its performances for distinguishing *Pf*-SM and *Pf*-UM patients were lower than those reported for apolipoprotein E, retinol-binding protein 4 (RBP4), and Hpx.	[78,94]
**Retinol-binding protein 4†**	This protein is a protein of the lipocalin family and well known for its crucial role in the transport of retinol (vitamin A) in the bloodstream.	RBP 4 exhibited good prognosis potential for discrimination between *Pf*-SM and *Pf*-UM patients.	[78,95]
**Plasminogen†**	Protein playing a role in the coagulation process and other physiological and pathological processes (e.g. angiogenesis, inflammation, oncogenesis).	Its plasma level was altered depending on the severity of malaria. Its performances for distinguishing *Pf*-SM and *Pf*-UM patients were lower than those reported for apolipoprotein E, RBP4, and Hpx.	[78,96]
**Apolipoproteins†**	Group of proteins whose pathological increase in the body is related to the genesis of vascular diseases as arteriosclerosis and atherosclerosis.	Apolipoprotein E exhibited good prognosis potential for discrimination between *Pf*-SM and *Pf*-UM patients.	[64,78,97]
**Endoglin**	Transmembrane glycoprotein expressed on ECs operating as a co-receptor for several ligands of the TGF-β family. This protein is also a recognized marker of angiogenesis.	Endoglin levels were statistically higher in *Pf*-CM and *Pf*-SM as compared to *Pf*-UM and HC. No statistical difference between *Pf*-SM and *Pf*-CM.	[98]
**B-cell-activating factor (BAFF)**	Cytokine of the tumor necrosis family playing an important role in the differentiation and survival of B cells throughout their different developmental stages.	Significant progressive increase in BAFF concentration with disease severity (*p* = 0.0001). Children whose BAFF plasma levels were above the median BAFF plasma level were at a higher risk of being admitted to hospital either with *Pf*-UM or *Pf*-SM.	[99]
**Complement fractions (C3, C4, Bb, C4d, iC3b, and SC5b-9)**	Complement is a set of a small proteins produced by the liver and that are found in the bloodstream as inactive precursors. The proteins play a role in several immune processes such as inflammation,and phagocytosis.	On admission, SC5b-9, and C4d were significantly higher in *Pf*-SM compared to HC. C3 and iC3b were significantly lower in *Pf*-SM compared to HC. No difference for C4 between *Pf*-SM and HC.	[100]
**BDCA3-positive dendritic cells**	Dendritic cells are antigen-presenting cells that are crucial for the initiation of adaptive immune responses.	The frequency of BDCA3 dendritic cells was significantly increased in *Pf*-SM compared to HC but similar between SM and CP.	[101]
**Vγ9 Vδ2 γδ T cells**	Subtype of γδ T cells which acts as a bridge between the innate and adaptive immune response, and whose effector functions include the production of cytokines and direct cytotoxicity to pathogens/infected cells.	Vγ9 Vδ2 γδ T cells were lower in children presenting with *Pf*-UM and *Pf*-CM than in HC patients but did not vary between *Pf*-UM and *Pf*-CM patients (*p* = 0.224).	[102]
**Asymmetric dimethylarginine (ADMA)**	Biomolecules working as an endogenous inhibitor of nitric oxide synthase and thus contributing to endothelial dysfunction.	Pre-treatment ADMA levels were significantly reduced in *Pf*-SM and *Pf*-UM as compared HC. ADMA levels significantly increased in *Pf*-SM patients after commencement of antimalarial therapy.	[103]
**Syndecan-1 and glycosaminoglycans**	These are products of the degradation of glycocalyx and thus are considered as markers of its degradation.	These were significantly higher in *Pk*-SM as compared to *Pk*-UM and HC. These were significantly higher in *Pv*-SM as compared to *Pv*-UM and HC. They were significantly correlated with other putative SM biomarkers (e.g. Ang-2, ICAM-1, E-selectin, osteoprotegerin, ADMA).	[104]
***pvcrt-o*****and*pvmdr1***	These two parasite genes are thought to be involved in the resistance of *Pv* parasites to antimalarial drugs such as chloroquine, as clearly established with their orthologues in *Pf* parasites.	The level of expression of these two genes was higher in *Pv*-SM compared to *Pv*-MM patients.	[105,106]

†Identified using proteomics analysis in some studies.

ADMA: asymmetric dimethylarginine, ARDS: acute respiratory distress syndrome, BAFF: B-cell-activating factor, CI: confidence interval, CM: cerebral malaria, ECs: Endothelial cells, CP: convalescent patients, *crt-o*: chloroquine resistance transporter-orthologue, Epo: erythropoietin, EVs: extracellular vesicles, Gal-9: galectin 9, GrzB: granzyme B, Hb : hemoglobin, HC: healthy control, Hpx: hemopexin, *mdr1*: multidrug resistance gene, MP: microparticles, OR: Odds ratio, PA: pantetheinase, *Pf*: *Plasmodium falciparum*, *Pk*: *Plasmodium knowlesi*, *Pv*: *Plasmodium vivax*, RBC: red blood cell, RBP4: retinol-binding protein 4, RD: respiratory distress, SM: severe malaria, SMA: severe malaria, anemia, TGF: transforming growth factor, TNF: tumor necrosis factor, UM: uncomplicated malaria, vWF: von Willebrand factor.

## Conclusion

The present review concluded that the identification and validation of biomarkers of SM are still in an early phase. Some candidate biomarkers seem to be promising for SM prognosis, but further studies are still required to confirm their prognostic value. Also, this review presented different aspects for which the prognostic value of candidate biomarkers was evaluated and summarized these findings from different studies. The authors also point out the need for further studies/research in the identification of predictive and therapeutic biomarkers, which are other critical links to be addressed for efficient control of SM. Although prognostic marker validation is relatively easy, more stringent criteria are required for the validation of predictive and therapeutic biomarkers for SM. Also, the concurrent and mixed *Plasmodium* infections are major obstacles for a better understanding of the relationship between the candidate biomarker and SM, and their evaluation as a potential biomarker needs to be carried out in further research studies. The advent of omics technologies would also be helpful in enhancing the identification and validation of clinical SM biomarkers, but affordable alternative assay systems need to be developed for translation in the clinical context of malaria.

## Supplementary Material

Supplemental Material

## Data Availability

All data included in this study are fully available from the corresponding author on reasonable request.

## Abbreviations

AMDA: Asymmetric dimethylarginine
AHSG: Alpha-2-Heremans-Schmid Glycoprotein
Ang: Angiopoietin
ARDS: Acute respiratory distress syndrome
AUC: Area under the curve
BAFF: B-cell-activating factor
CAMs: Cell adhesion molecules
CCL: Chemokine ligand
CHI3L1: Chitinase-3-like 1 protein
CI: Confidence interval
CM: Cerebral malaria
CMNS: Cerebral malaria nonsurvivors
CMS: Cerebral malaria survivors
CP: Convalescent patients
CRP: C-reactive protein
*crt-o*: Chloroquine resistance transporter-Orthologue
CSF: Cerebrospinal fluid
DNA: Deoxyribonucleic acid
ECs: Endothelial cells
ELISA: Enzyme-linked immunosorbent assay
EPCR: Endothelial protein C receptor
Epo: Erythropoietin
EVs: Extracellular vesicles
Gal-9: Galectin-9
GrzB: Granzyme B
Hb: Hemoglobin
HC: Healthy control
Hct: Hematocrit
HMGB1: High-mobility group box protein 1
Hp: Haptoglobin
Hpx: Hemopexin
HRP2: Histidine-rich protein 2
IFN: Interferon
IL: Interleukin
IP-10: 10-kDa INF gamma-induced protein
LDR-FMA: Ligase detection reaction-fluorescent microsphere assay
LM: Light microscopy
LODS: Lambaréné Organ Dysfunction Score
*mdr1*: Multidrug resistance gene 1
MM: Mild malaria
MP: Microparticles
NLR: Negative likelihood ratio
NPV: Negative predictive value
OR: Odd ratio
PA: Pantetheinase
PD: Parasite density
PCM/PCN: Pigment-containing monocytes/neutrophils
PCR: Polymerase chain reaction
PCT: Procalcitonin
*Pf*: *Plasmodium falciparum*
PfEMP1: *Pf* erythrocyte membrane protein 1
*Pk*: *Plasmodium knowlesi*
*Pm*: *Plasmodium malariae*
*Po*: *Plasmodium ovale*
*Pv*: *Plasmodium vivax*
PLR: Positive likelihood ratio
PPV: Positive predictive value
PWD: Platelet width distribution
QBC: Quantitative buffy coat
RBCDW: Red blood cell distribution width
RBP4: Retinol-binding protein 4
RD: Respiratory distress
RDT: Rapid diagnostic test
RN: Retinopathy negative
RNA: Ribonucleic acid
RP: Retinopathy positive
ROC: Receiver operating characteristic
SBP: Systolic blood pressure
sICAM-1: Soluble intercellular cell adhesion molecule 1
Se: Sensitivity
SEA: South East Asia
SERPINA3: Serpin peptidase inhibitor clade A member 3
SM: Severe malaria
SMA: Severe malarial anemia
Sp: Specificity
sSA: Sub-Saharan Africa
sTie-2: Soluble cognate receptor
suPAR: Soluble urokinase-type plasminogen activator
TGF: Transforming growth factor
TNF: Tumor necrosis factor
UM: Uncomplicated malaria
VCAM-1: Vascular adhesion molecule 1
VEGF: Vascular endothelial growth factor
vWF: von Willebrand factor
WHO: World Health Organization
