# Supplementary material for: The spectrum of clinical biomarkers in severe malaria and new avenues for exploration
Source: Virulence. 2022 Aug 29;13(1):634–53. doi: 10.1080/21505594.2022.2056966 (PMC9427047; doi:10.1080/21505594.2022.2056966)
Supplement: Supplemental Material [file KVIR_A_2056966_SM1249.zip › supplementary/Clean_ Supplemental material 2.docx]

**Supplemental material 2**. Performances of the proposed biomarkers for severe *Pf* malaria

**Table S2**. (references are presented at the end of this supplemental material)

| **Biomarker** | **Origin** | **Nature** | **Endpoint** | **Country** | **Major findings from the study** | **Measurement (Body fluid)** | **Ref.** |
| --- | --- | --- | --- | --- | --- | --- | --- |
| Haptoglobin |  |  | Respiratory distress | The Gambia, Kenya | - The combination of lipocalin 2 (Lpc-2) and Hp were effective in discriminating between pneumonia and RD; Hp + Lpc-2 (AUC = 0.99 in The Gambia, AUC = 0.86 in Kenya). | Mass spectrometry–based proteomic study + ELISA (Plasma) | [1] |
|  |  |  | Malaria severity | India | - Its plasma level was altered depending on the severity of malaria; - Its performances for distinguishing *Pf*-SM and *Pf*-UM patients were lower than those for apolipoprotein E, retinol binding protein 4 (RBP4) and Hemopexin (Hpx). | Proteome analysis + ELISA (Serum) | [2] |
| Circulatory complement‑lysis inhibitor or clusterin (CLI/CLU) | Host | Protein | CM | Nigeria | - CLI level was low at hospital admission in the CM group, and returned to normal with convalescence; - CM *vs* UM (AUC = 0.97, 95% CI 0.9335-1.007, *p* < 0.0001); - CM *vs* HC (AUC = 0.94, 95% CI 0.8764-1.006, *p* < 0.0001). | ELISA (Plasma) | [3] |
| Pigment-containing cells (neutrophils, eosinophils, granulocytes, monocytes) |  |  | Mortality | Thailand | - PCN and PCM counts were higher in deceased than in survivors; - These two pigment-containing phagocytes had higher Sp and Se values than parasitemia: Optimal cut-off [PCN : >4/100, Se = 73 % (56-85), Sp = 77 % (71-82); PCM : >5/30, Se = 70 % (53-83), Sp = 64 % (58-70)]. | Light microscopy | [4] |
|  |  |  | Mortality | The Gambia, Gabon, Ghana, Kenya, Malawi | - PCN, PCE and PCG were significantly higher in died than in survivors; - The predictive ability of pigmented cells was low, as measured by the change in the AUC of logistic regression models. | Flow cytometry (Whole blood) | [5] |
|  |  |  | Malaria severity | Sudan | - No difference in PCM count between SM and CM (*p* = 0.052); - UM *vs* SM (AUC = 0.68, *p* = 0.0015; Cut-off = 5.5 % with Se = 68.2 % and Sp = 65.2 %). | Light microscopy (Whole blood) | [6] |
| *var* gene | Parasite | DNA | Malaria severity | India | - *var* group A was significantly higher in SM and CM (*p* = 0.007 and *p* = 0.011) than *var* group C; - *var* group 1 (cys2/MFK*) was significantly higher in CM patients (*p* < 0.0001) compared to both SM and MM; - *var* group 3 was higher in MM *vs* CM (*p* = 0.031); - In MM (*var* group A had a significant higher AUC compared to groups B (AUC = 0.92 *vs* AUC = 0.51, *p* = 0.011971) and C (AUC = 0.92 *vs* AUC = 0.52, *p* = 0.0032925); - The performances of *var* groups A, B and C were similar for SM and CM, with the exception of *var* A and *var* B in SM patients (AUC = 0.66, 95% CI 0.54-0.79 for group A and 0.49, 95% CI 0.35-0.62 for group B, *p* = 0.037738). | Genotyping/Cloning (Whole blood) | [7] |
|  |  |  | Malaria severity | Tanzania, Malawi, India | - The upregulation of group A and DC8 predict SM in adults and children; - UM *vs* SM: India (AUC = 0.72, 95% CI 0.62-0.83), Malawi (AUC = 0.75, 95% CI 0.65-0.84), and Tanzania (AUC = 0.81, 95% CI 0.73-0.90). | n.a | [8]¶ |
| C-reactive protein | Host | Protein | Mortality | Uganda | - CRP was significantly higher in CM and SMA compared to UM; - AUC = 0.72, Cut-off > 43.1 ng/mL had Se, Sp, PPV and NPV values of 56.5 %, 82.5 %, 16.3 % and 96.9 %. | ELISA (Serum) | [9] |
|  |  |  | Malaria severity | The Netherlands | - CRP was significantly higher in *Pf*-SM compared to *Pf*-UM and non-*Pf* UM; - SM *vs* UM: AUC = 0.84, *p* = 0.0237; Cut-off ≥ 155 mg/L with a Se = 79 %, Sp = 80 %, PPV = 35 % and NPV = 97 %. | ELISA (Serum) | [10] # |
|  |  |  | Malaria severity | The Netherlands | - CRP was significantly higher in *Pf*-SM compared to *Pf*-UM and non-*Pf* UM; - SM *vs* UM: Cut-off ≥ 142 mg/L with a Se = 76 %, Sp = 75 %, PPV = 35 % and NPV = 95 %). | ELISA (Serum) | [11] # |
|  |  |  | Malaria severity | India | - CRP was significantly higher in SM compared to UM and HC (*p* = 0.013); - SM *vs* UM: AUC = 0.786, Cut-off of 1.85 mg/dL had Se = 71.4 % and Sp = 68.7 %. | Turbidimetric immunoassay (Serum) | [12] |
|  |  |  | Malaria severity | India | - SM *vs* UM: Cut-off of 2 ng/mL had Se = 100 %, Sp = 63.16 %, PPV = 85.12 % and NPV = 100 %; AUC = 0.915 (95% CI, 0.687 to 0.908). | BRAHMS PCT-Q® test  (Serum) | [13] |
|  |  |  | Malaria severity | Italy | - CRP level of 100 mg/dL had Se = 89 %, Sp = 57 %, PPV = 47 % and NPV = 92%. | Automated immunofluorescent assays (Plasma) | [14] # |
|  |  |  | CM | Germany | - CM *vs* UM: AUC = 0.771, 95% CI 0.645 – 0.896, *p* = 0.003 | In-house tests (Plasma) | [15] |
| CK-MB | Host | Protein | Malaria severity | India | - CK-MB was significantly higher in SM compared to UM and HC; - SM *vs* CM: AUC = 0.823, 95% CI 0.723-0.922. | ELISA and Spectrometry-based Kits | [16] |
| CKD-EPI eGRF | Host | Biological process | Malaria severity | India | - CKD-EPI eGRF was significantly higher in SM compared to UM and HC; - SM *vs* CM: AUC = 0.760, 95% CI 0.640-0.879. | - | [16] |
| Caveolin 1 | Host | Protein | Malaria severity | India | - Caveolin 1 was significantly higher in SM compared to UM and HC; - SM *vs* CM: AUC = 0.73, 95% CI 0.613-0.862. | ELISA (Plasma) | [16] |
| pLDH | Parasite | Protein | Malaria severity | India | - pLDH was significantly higher in SM compared to UM; - SM *vs* CM: AUC = 0.790, 95% CI 0.606-0.974. | ELISA (Plasma) | [16] |
| Procalcitonin | Host | Protein | Malaria severity | Germany | - Pre-treatment PCT concentrations were closely correlated with parasitemia; - PCT levels were highest in SM patients; - Discriminant analysis (Jackknife method): PCT correctly classified non-survivors in 100% and survivors in 90 %. | Immunoluminometric assay (Serum) | [17] # |
|  |  |  | Malaria severity | The Netherlands | - *Pf*-SM patients had the highest median PCT levels (*p* < 0.0001); - At a cut-off point of 10 ng/mL had a Se = 67 %, Sp = 94 %. | BRAHMS PCT-Q® test  (Serum) | [18] # |
|  |  |  | Malaria severity | The Netherlands | - On admission, PCT levels were significantly higher in SM compared to UM; - AUC = 0.78; 95% CI 0.66-0.91; - A cut-off point of 10.0 ng/mL, PCT had a PPV and NPV of 30 % and 100 %, respectively. | ELISA (Serum) | [19] # |
|  |  |  | Mortality | Uganda | - PCT was significantly higher in fatal cases; - Survivors *vs* fatal cases (AUC = 0.72, 95% CI 0.62 - 0.80; Cut-off of > 43.1 ng/mL had a Se = 56.5 %, Sp = 82.5 %, PPV = 16.3 % and NPV = 96.9 %). | ELISA (Serum) | [9] |
|  |  |  | Malaria severity | The Netherlands | - PCT was significantly higher in *Pf*-SM compared to UM and non-*Pf*-UM; - AUC = 0.76, *p* = 0.6479; - Cut-off ≥ 0.9 ng/mL with a Se = 100 %, Sp = 51 %, % PPV = 28 % and NPV = 100 %). | ELISA (Serum) | [10] # |
|  |  |  | Malaria severity | India | - SM *vs* UM: An optimal cut-off of 2 ng/mL had Se = 100 %, Sp = 63.16 %, PPV = 85.12 % and NPV = 100 %; AUC = 0.915 (95% CI, 0.687 - 0.908). | BRAHMS PCT-Q® test (Serum) | [13] |
|  |  |  | Malaria severity | Italy | - PCT was correlated with parasitaemia; - PCT was higher in SM compared to UM; - PCT levels > 5 ng/mL had a Se = 67 %, Sp = 86 %, PPV = 67 %, NPV = 86 %. | Automated immunofluorescent assays (Plasma) | [14] |
| Angiopoeitins-1 and 2 | Host | Protein | Discrimination between CM and non-CM SM from UM | Thailand | - Significant decrease in Ang-1 in CM compared to SM and UM; - UM *vs* SM (Ang-2: AUC = 0.763, *p* < 0.001, Cut-off =1.43 ng/mL with Se = 69.4 %, Sp = 78.6 %, LR+ = 3.241 and LR- = 0.389; Ang-1: AUC = 0.884, *p* < 0.001, Cut-off = 12.38 ng/mL with Se = 86.1 %, Sp = 85.2 %, LR+ = 6.028 and LR- = 0.162; Ang-2/Ang-1 ratio: AUC = 0.857, *p* < 0.001, Cut-off 0.082 with Se = 80.6 %, Sp = 82.9 %, LR+ = 4.699 and LR- = 0.235); - UM *vs* CM (Ang-2: AUC = 0.772, *p* < 0.001, Cut-off 1.33 ng/mL with Se = 75.9 %, Sp = 77.1 %, LR+ = 3.319 and LR- = 0.313; Ang-1: AUC = 0.778, *p* < 0.001, Cut-off 15.86 ng/mL with Se = 71.3 %, Sp = 74.3 %, LR+ = 2.771 and LR- = 0.387; Ang-2/Ang-1 ratio: AUC = 0.820, *p* < 0.001, Cut-off 0.080 with Se = 78.2 %, Sp = 82.9 %, LR+ = 4.559 and LR- = 0.264); - SM *vs* CM (Ang-2: Cut-off 3.14 ng/mL with Se = 47.2 %, Sp = 64.4 %, LR+ = 1.325 and LR- = 0.820; Ang-1: Cut-off 11.21 ng/mL with Se = 72.2 %, Sp = 65.5 %, LR+ = 2.094 and LR- = 0.424; Ang-2/Ang-1 ratio: Cut-off 0.448 with Se = 44.4 %, Sp = 82.8 %, LR+ = 2.578 and LR- = 0.671). | ELISA (Whole blood) | [20] |
|  |  |  | Malaria severity | Nigeria | - Significant decrease in Ang-1 in SM and UM compared to HC; - Significant increase in Ang-2, and Ang-2/Ang-1 ratio in SM and UM compared to HC; - SM *vs* UM: Ang-1: AUC = 0.5046, *p* = 0.9480; Ang-2: AUC = 0.810, *p* < 0.0001; Ang-2/Ang-1 ratio: AUC = 0.723, *p* = 0.001479. | ELISA (Serum) | [21] |
|  |  |  | Mortality | Uganda | - Ang-2 was significantly higher in fatal cases; - Survivors *vs* fatal cases (Ang-2: AUC = 0.83, 95% CI 0.75 - 0.90; Cut-off of > 5.6 ng/mL with Se = 78.3 %, Sp = 78.8 %, PPV = 18.2 % and NPV = 98.4 %). | ELISA (Serum) | [9] |
|  |  |  | RP-CM mortality | Malawi | - (Ang-1: AUC = 0.56, 95% CI 0.47-0.56, *p* = 0.20; Ang-2: AUC = 0.71, 95% CI 0.63-0.78, *p* < 0.0001). | ELISA (Plasma) | [22] |
|  |  |  | CM | Thailand,  Uganda | - In both populations, Ang-1 levels were significantly decreased and Ang-2 levels were significantly increased in CM *versus* UM or HC (*p* < 0.001); - Ang-1 (Thailand: AUC = 1, *p* < 0.001; Uganda: AUC = 0.785, *p* < 0.001); - Ang-2 (Thailand: AUC = 0.835, *p* < 0.001; Uganda; AUC = 0.688, *p* < 0.001); - Ang-2/Ang-1 ratio (Thailand: AUC = 1, *p* < 0.001; Uganda: AUC = 0.779, *p* < 0.001); - Optimal cut-off [Thailand: Cut-off of 21.26 ng/mL had a Se = 100 %, Sp = 100 % (Ang-1); cut-off of 3.04 ng/mL had a Se = 72 % and Sp = 84 % (Ang-2), and cut-off of 0.131 with Se = 100 % and Sp = 100 % (Ang-2/Ang-1 ratio); Uganda: Cut-off of 15.05 ng/mL had a Se = 70 %, Sp = 75 % (Ang-1); cut-off of 0.39 ng/mL had a Se = 83 % and Sp = 60 % (Ang-2), and cut-off of 0.052 had a Se = 73 % and Sp = 70 % (Ang-2/Ang-1 ratio)]. | ELISA (Plasma) | [23] |
|  |  |  | Severity,  Clinical recovery, Mortality | Uganda | - The Ang-2 levels were also associated with post-discharge mortality (*p* < 0.0001); - The model including LODS alone had an AUC of 0.77 (95% CI 0.67–0.88); - Model including Ang-2 was significantly better than LODS alone at predicting in-hospital mortality with AUC = 0.85 (95% CI 0.79–0.90; *p* = 0.03). | ELISA (Plasma) | [24] |
|  |  |  | RP-CM mortality | Malawi | - RP-CM vs RN-CM : Ang-1 [AUC = 0.64, 95% CI 0.51-0.78, *p* = 0.046; A cut-off of 3.2 had Se = 68%, Sp = 44%, PLR = 1.2, and NLR = 0.70]; Ang-2 [AUC = 0.77, 95% CI 0.65-0.89, *p* < 0.0001 ; A cut-off of 6.2 had Se = 71%, Sp = 72%, PLR = 2.6, and NLR = 0.40]; Ang-2/Ang-1 [AUC = 0.74, 95% CI 0.60-0.87, *p* = 0.001 ; A cut-off of 2.0 had Se = 74%, Sp = 66%, PLR = 2.1, and NLR = 0.40]. | ELISA (Plasma) | [25] |
| Soluble cognate receptor (sTie-2) | Host | Protein | RP-CM mortality | Malawi | - RP-CM vs RN-CM : AUC = 0.83, 95% CI 0.73-0.93, *p* < 0.0001 ; A cut-off of 56 had Se = 74%, Sp = 72%, PLR = 2.7, and NLR = 0.36. | ELISA (Plasma) | [25] |
|  |  |  | RP-CM mortality | Malawi | - Survivors *vs* fatal cases : AUC = 0.64, 95% CI 0.55-0.73, *p* = 0.003. | ELISA (Plasma) | [22] |
| von Willebrand factor propeptide | Host | Protein | RP-CM mortality | Malawi | - RP-CM vs RN-CM : AUC = 0.71, 95% CI 0.58-0.85, *p* = 0.0005; A cut-off of 51 had Se = 76%, Sp = 62%, PLR = 2.0, and NLR = 0.38. | ELISA (Plasma) | [25] |
| von Willebrand factor (vWF) | Host | Protein | RP-CM mortality | Malawi | - RP-CM vs RN-CM : AUC = 0.58, 95% CI 0.42-0.73, *p* = 0.305; A cut-off of 310 had Se = 68%, Sp = 58%, PLR = 1.7, and NLR = 0.54. | ELISA (Plasma) | [25] |
|  |  |  | CM | Uganda | - CM *vs* UM: AUC = 0.67, 95% CI: 0.58-0.75 | ELISA (Serum) | [26] |
| Vascular endothelial growth factor (VEGF) | Host | Protein | RP-CM mortality | Malawi | - RP-CM vs RN-CM : AUC = 0.65, 95% CI 0.52-0.79, *p* = 0.040; A cut-off of 0.25 had Se = 74%, Sp = 58%, PLR = 1.7, and NLR = 0.46. | ELISA (Plasma) | [25] |
| Chitinase-3-like 1 | Host | Protein | Malaria severity, Mortality | Uganda | - CHI3L1 was significantly higher in children with CM and SMA compared to UM (*p* < 0.001); - CHI3L1 levels were higher among children with CM and SMA who subsequently went on to die of their infection, compared to survivors (*p* < 0.01); - No difference between CM and SM for CHI3L1 levels; - On analysis of SM cases (CM + SMA), a good discriminatory ability between fatalities and survivors (AUC = 0.84, 95% CI 0.76-0.92); A cut-point of 179.1 ng/mL had Se = 91.3 % and Sp = 67.5 % for predicting mortality. | ELISA (Plasma) | [27] |
| High mobility group box 1 (HMGB1) | Host | Host | Malaria severity, Mortality | Uganda | - At admission, HMGB1 levels were significantly higher i) in SM compared to UM, and ii) in died compared to survivors; - AUC = 0.72, 95% CI 061-0.83 (fatal *vs* non-fatal). | ELISA (Plasma) | [28] |
| Elastase-2 | Host | Protein | CM | Germany | - CM *vs* UM: AUC = 0.699, 95% CI 0.569 – 0.829, *p* = 0.029 | ELISA (Plasma) | [15] |
| D-dimers | Host | Protein | CM | Germany | - CM *vs* UM: AUC = 0.775, 95% CI 0.643 – 0.915, *p* = 0.019 | In house assay (Plasma) | [15] |
| Fibrinogen | Host | Protein | CM | Germany | - CM *vs* UM: AUC = 0.482, 95% CI 0.287 – 0.676, *p* = 0.842 | In house assay (Plasma) | [15] |
| Myeloperoxidase (MPO) | Host | Protein | CM | Germany | - CM *vs* UM: AUC = 0.752, 95% CI 0.604 – 0.900, *p* = 0.006 | ELISA (Plasma) | [15] |
| sVCAM-1 | Host | Protein | CM | Germany | - CM *vs* UM: AUC = 0.752, 95% CI 0.604 – 0.900, *p* = 0.006 | ELISA (Plasma) | [15] |
|  |  |  | CM | Uganda | - CM *vs* UM: AUC = 0.59, 95% CI: 0.50-0.68 | ELISA (Serum) | [26] |
| sICAM-1 | Host | Protein | Mortality | Uganda | - ICAM-1 was significantly higher in fatal cases; - Survivors *vs* fatal cases (AUC = 0.84, 95% CI 0.75 - 0.90; Cut-off of > 645.3 ng/mL with Se = 87 %, Sp = 75 %, PPV = 17.4 % and NPV = 99.0 %). | ELISA (Serum) | [9] |
|  |  |  | CM | Germany | - CM *vs* UM: AUC = 0.784, 95% CI 0.650 – 0.919, *p* = 0.003 | ELISA (Plasma) | [15] |
|  |  |  | RP-CM mortality | Malawi | - RP-CM vs RN-CM : AUC = 0.73, 95% CI 0.59-0.86, *p* = 0.006; A cut-off of 1000 had Se = 71%, Sp = 69%, PLR = 2.3, and NLR = 0.42. | ELISA (Plasma) | [25] |
|  |  |  | CM | Uganda | - CM *vs* UM: AUC = 0.54, 95% CI: 0.45-0.63 | ELISA (Serum) | [26] |
|  |  |  | Malaria severity,  Mortality | Uganda | - Survivors *vs* fatal cases: The combination CHI3L1 + Ang-2 + sICAM-1 had a Se = 95.7 %, Sp = 81.3 %, PPV = 23.6 %, and NPV = 99.7 %. | ELISA (Plasma) | [27] |
| PfHRP2 | Parasite | Protein | CM | Malawi | - Elevated pPfHRP2 allow distinction between RP and RN patients with CM; - CM-RP *vs* CM-RN: pPfHRP2 level of >1700 ng/mL (Cross-sectional study: Se = 98 %, Sp = 94 %, AUC = 0.98; Autopsy study: Se = 90 %, Sp = 87 %, AUC = 0.90). | ELISA (Plasma) | [29] |
|  |  |  | Progression to CM | Malawi | - An HRP2 concentration of 3500 ng/mL predicted subsequent clinical deterioration with a Se = 88% and Sp = 88%; - The AUC was 0.925 (progression to CM). | ELISA (Plasma) | [30] |
|  |  |  | CM (Distinction between CM and other encephalopathies) | Kenya | - An HRP2 level of >0 U/mL had a MAF of 93% for CM, with a MAF of 97% observed for HRP2 levels of ≥10 U/mL (the level of the best combined Se and Sp). | ELISA (Plasma) | [31] |
|  |  |  | CM (RN-CM *vs* other forms of SM, and AM) | Uganda | - HRP2 was higher in CM-RP compared to CM-RN patients (*p* = 0.006); - HRP2 is higher in CM-RN patients compared to SMA and AM; - CM-RP *vs* CM-RN: AUC = 0.61, 95% CI 0.53-0.68. | ELISA (Plasma) | [32] |
|  |  |  | CM, CM presentation, Mortality | Malawi | - UM *vs* CM (AUC = 0.895, 95% CI 0.853-0.940, *p* ≤ 0.0001); - RP *vs* RN (AUC = 0.697, 95% CI 0.596-0.798, *p* = 0.0003); - RP survivors *vs* RP fatal (AUC = 0.695, 95% CI 0.518-0.877, *p* = 0.013). | ELISA (Plasma) | [33]& |
| Red blood cell distribution | Host | Cellular | Malaria severity | Sudan | - RBCDW was significantly higher in SM compared to UM (*p* = 0.002); - RDCW (AUC = 0.65, Se = 71.5 %, Sp = 61.2 %). | Haematological analyser | [6] |
| Platelet distribution width | Host | Cellular | Malaria severity | Sudan | - PDW was significantly higher in SM compared to UM (*p* < 0.0001); - PDW (AUC = 0.69, Se = 80.1 %, Sp = 66.3 %). | Haematological analyser | [6] |
| Oxylipin and endocannabidome metabolites | Host | Lipid | Malaria severity | Rwanda | - Oxylipin and endocannabidome metabolites were significantly higher in UM and SM compared to HC; - Thromboxane B2 was the only compound that survived the Bonferroni cut off value (*p* = 0.00125) [SM *vs* HC: AUC = 0.84, 95% CI 0.71-0.96, *p* = 0.0002246; UM *vs* HC (AUC = 0.75, 95% CI 0.60-0.91, *p* = 0.0007245]. | The stable isotope dilution method (Plasma) | [34] |
| sTREM-1 | Host | Protein | Mortality | Uganda | - Survivors *vs* fatal cases :AUC = 0.76, 95% CI 0.66 - 0.84; Cut-off of > 289.9 ng/mL with Se = 95.7 %, Sp = 43.8 %, PPV = 9.3 % and NPV = 99.4 %. | ELISA (Serum) | [9] |
| sCD14 | Host | Protein | Malaria severity | Cameroon | - AM *vs* HC (AUC = 0.842, 95% CI 0.735-0.939); AM *vs* UM (AUC = 0.876, 95% CI 0.817-0.936), CM *vs* UM (AUC = 0.100, 95% CI 0.047-0.154) | ELISA (Serum) | [35] |
| sCD163 | Host | Protein | Malaria severity | Cameroon | - AM *vs* HC (AUC = 0.959, 95% CI 0.922-0.996); AM *vs* UM (AUC = 0.499, 95% CI 0.395-0.602), CM *vs* UM (AUC = 0.308, 95% CI 0.213-0.414). | ELISA (Serum) | [35] |
| suPAR | Host | Protein | Malaria severity | Cameroon | - AM *vs* HC (AUC = 0.45, 95% CI 0.337-0.632); AM *vs* UM (AUC = 0.958, 95% CI 0.922-0.994), CM *vs* UM (AUC = 0.678, 95% CI 0.575-0.782). | ELISA (Serum) | [35] |
| Pentraxin 3 | Host | Protein | Malaria severity | Cameroon | - AM *vs* HC (AUC = 0.233, 95% CI 0.135-0.331); AM *vs* UM (AUC = 0.979, 95% CI 0.958-0.999), CM *vs* UM (AUC = 0.401, 95% CI 0.297-0.504). | ELISA (Serum) | [35] |
| Neopterin | Host | Protein | Malaria severity | The Netherlands | - On admission, neopterin levels were significantly higher in SM compared to UM; - AUC = 0.85; 95% CI 0.76-0.94; an optimal cut-off point of 10.0 ng/mL with a Se, Sp, PPV and NPV of 93 %, 67%, 38 % and 98 %, respectively. | ELISA (Serum) | [19]# |
|  |  |  | Malaria severity | Cameroon | - AM *vs* HC (AUC = 0.857, 95% CI 0.760-0.954); AM *vs* UM (AUC = 0.246, 95% CI 0.149-0.343), CM *vs* UM (AUC = 1, 95% CI n.a). | ELISA (Serum) |  |
| Hyponatremia/  Sodium | Host | Signature of biological process | Malaria severity | The Netherlands | - Prevalence and severity of hyponatremia were greatest in *Pf*-SM; - Admission serum sodium cut-off of < 133 mmol/L had a Se = 69 % and Sp = 76 % for predicting SM; - UM *vs* SM: AUC = 0.72. | Indirect potentiometry (Serum) | [36]# |
|  | Host | Ion | Malaria severity | The Netherlands | - Sodium significantly decreased in *Pf*-SM compared to UM and non-*Pf* UM; - UM *vs* SM: AUC = 0.72, *p* = 0.4289 - Cut-off of < 132 mmol/L had a Se = 72 %, Sp = 72 %, PPV = 26 %, NPV = 95 %. | Ionogram (Serum) | [10]# |
| Copeptin | Host | Protein | Malaria severity | The Netherlands | - Copeptin was significantly higher in *Pf*-SM compared to *Pf*-UM and non-*Pf* UM; - Cut-off of 21 pmol/L had a Se = 60 %, Sp = 73 %, PPV = 31 %, and NPV = 93 %; - AUC = 0.66, *p* = n.a. | ELISA (Serum) | [10]# |
|  |  |  | CM | Germany | - CM *vs* UM: AUC = 0.596, 95% CI 0.420 – 0.771, *p* = 0.293 | Immunofluorescence assay (Plasma) | [15] |
| Cell-free DNA (cfDNA) | Host/Parasite | DNA | CM, Mortality | Malawi | - Total cfDNA was significantly higher in SM compared to UM (*p* < 0.0001) and HC (*p* < 0.0001); - Host cfDNA was the main contributor to total cfDNA; - CM *vs* UM (AUC = 0.694, 95% CI 0.630-0.760, *p* = 0.034 for total cfDNA; AUC = 0.772, 95% CI 0.730-0.833, *p* < 0.0001 for host cfDNA; and AUC = 0.760, 95% CI 0.678-0.842, *p* < 0.0001 for parasite cfDNA); - RP *vs* RN (AUC = 0.633, 95% CI 0.529-0.763, *p* = 0.015 for total cfDNA; AUC = 0.715, 95% CI 0.609-0.821, *p* = 0.0001 for host cfDNA; and AUC = 0.745, 95% CI 0.604-0.825, *p* = 0.0001 for parasite cfDNA); - Survivors *vs* fatal cases among the CM-RP cases (AUC = 0.779, *p* = 0.0006, for total cfDNA; AUC = 0.720, *p* = 0.001 for host cfDNA; and AUC = 0.420, *p* = ns, for parasite cfDNA. | Fluorescence assay (Plasma) | [33] |
| Chemokines: platelet factor-4 (CXCL4), Fractalkine (CX3CL1), Monokine induced by gamma (CXCL9), and interferon-γ inducible protein-10 (CXCL10) | Host | Protein | Malaria severity, mortality | India | - Levels of CXCL4 and CXCL10 were significantly elevated in CM fatal patients (*p* < 0.05) when compared with HC, MM and CM survivors; - CMNS *vs* MM (AUC = 1 for CXCL4 and CXCL10 respectively); - CMS *vs* MM (AUC = 0.35 and 0.78 for CXCL4 and CXCL10, respectively); - CMNS *vs* CMS (AUC = 1 for CXCL4 and CXCL10 respectively). | ELISA (Plasma) | [37] |
|  |  |  | Malaria severity | Cameroon | - AM *vs* HC (AUC = 0.985, 95% CI 0.956-1.000); AM *vs* UM (AUC = 0.314, 95% CI 0.218-0.410), CM *vs* UM (AUC = 0, 95% CI n.a) for CX3CL1 | ELISA (Serum) | [35] |
|  |  |  | Malaria severity | Cameroon | - AM *vs* HC (AUC = 0.788, 95% CI 0.666-0.910); AM *vs* UM (AUC = 0.490, 95% CI 0.387-0.593), CM *vs* UM (AUC = 0.566, 95% CI 0.431-0.702) for CXCL9. | ELISA (Serum) | [35] |
| Carboxyhemoglobin | Host | Protein | Malaria severity, Mortality | Indonesia | - Carboxyhemoglobin was significantly higher in SM and SS compared to HC and MM (*p* < 0.001); - Carboxyhemoglobin was significantly higher SM survivors compared to SM fatal (*p* = 0.02); - There was no significant change with carboxyhemoglobin levels with clinical recovery and time; - SM survivors *vs* SM fatal (AUC = 0.73, 95% CI 0.52-0.97). | Co-oximetry | [38] |
| Cytokines (Interleukins, TNF-α, IFN) | Host | Protein | CM | Thailand,  Uganda | - TNF- α was significantly lower in HC compared to UM and CM (*p* < 0.0001), but no difference UM *vs* CM - UM *vs* CM (Thailand: AUC = 0.834, 95% CI 0.713-0.955, *p* < 0.0001; Uganda: AUC = 0.557, 95% CI 0.453-0.661, *p* = 0.268). | ELISA (Plasma) | [23] |
|  |  | RNA | Malaria severity | India | - IL-1β, IL-1, IFN-γ, TGF-β discriminated between SM and UM as their level was upregulated in SM; - Modelling of data by logistic regression showed that IL-1b, TNF-a and the combination of IFN-g*IL-18 were predictive for SM, with the AUC of 0.986. | qPCR | [39] |
| Schizontaemia | Parasite | Cellular | Malaria severity | The Netherlands | - Schizontaemia was significantly higher in *Pf*-SM compared to *Pf*-UM and non-*Pf* UM; - Se = 53 %, Sp = 95 %, PPV = 67 %, NPV = 92 %. | Light microscopy | [11]# |
| 10 kDa INF gamma-induced protein (IP-10) | Host | Protein | Mortality | Uganda | - Survivors *vs* fatal cases: AUC = 0.80, 95C% 0.71-0.87, *p* < 0.01; - Survivors *vs* fatal cases: Cut-off of > 831.2 pg/mL with Se = 82.6 %, Sp = 85 %, PPV = 25 % and NPV = 98.8 %. | ELISA (Plasma) | [9] |
|  |  |  | RP-CM mortality | Malawi | - RP-CM vs RN-CM : AUC = 0.58, 95% CI 0.43-0.73, *p* = 0.312; A cut-off of 0.71 had Se = 66%, Sp = 54%, PLR = 1.4, and NLR = 0.64. | ELISA (Plasma) | [25] |
|  |  |  | Malaria severity,  Mortality | Uganda | - Survivors *vs* fatal cases: The combination CHI3L1 + IP-10 + sICAM-1 had a Se = 91.3 %, Sp = 83.8 %, PPV = 25.4 %, and NPV = 99.4 %; - Survivors *vs* fatal cases: The combination CHI3L1 + Ang-2 + IP-10 had a Se = 100 %, Sp = 91.3 %, PPV = 24.4 %, and NPV = 100 %. | ELISA (Plasma) | [27] |
| FMS-like tyrosine kinase-1 (sFlt-1) | Host | Protein | Mortality | Uganda | - AUC = 0.75, 95C% 0.65-0.83, *p* < 0.01; - Cut-off of > 1066.3 pg/mL with Se = 82.6 %, Sp = 57.5 %, PPV = 10.5 % and NPV = 98.2 %. | ELISA (Plasma) | [9] |
| Lactate | Host | Glucose product | Malaria severity | The Netherlands | - Lactate was significantly higher in *Pf*-SM compared to *Pf*-UM and non-*Pf* UM; - AUC = 0.74, *p* = 0.6546 - Cut-off of ≥ 1.6 mmol/L had a Se = 86 %, Sp = 56 %, PPV = 29 %, NPV = 95 %. | ELISA (Plasma) | [10]# |
|  |  |  | Malaria severity | The Netherlands | - Cut-off of ≥ 1.7 mmol/L had a Se = 80 %, Sp = 63 %, PPV = 39 %, NPV = 91 %. | ELISA (Plasma) | [11]# |
|  |  |  | Malaria severity | The Netherlands | - UM *vs* SM: AUC = 0.80, 95% CI 0.65-0.96 | ELISA (Plasma) | [19]# |
| *Pf* DNA levels |  |  | Malaria severity | Mozambique, Tanzania, Bangladesh, India | - UM *vs* SM: AUC = 0.834, *p* = 0.43 in children, and AUC = 0.789, *p* = 0.29 in adults; - The combination of *Pf* DNA and PfHRP2 levels improved the discrimination between UM and SM patients (AUC = 0.904, *p* = 0.004 in children, and AUC = 0.847, *p* = 0.003 in adults). | qPCR (Plasma) | [40]* |
| Platelet count | Host | Cellular | CM-related Retinopathy | Malawi | - RP *vs* RN (AUC = 0.75). | Haematological analyser | [29] |
|  |  |  | Malaria severity | Sudan | - Significant decrease in platelet in SM and UM compared to HC; - SM *vs* UM: AUC = 0.820, *p* < 0.0001, Cut-off of 200 × 10^3^ with a Se = 80.7 % and Sp = 75 %. | Haematological analyser | [6] |
|  |  |  | Malaria severity | Nigeria | - Significant decrease in platelet in SM and UM compared to HC; - SM *vs* UM: AUC = 0.9258, 95%CI 0.8557-0.9960, *p* < 0.0001. | Haematological analyser | [21] |
|  |  |  | CM, Mortality | Malawi | - UM *vs* CM (AUC = 0.932, 95% CI 0.900-0.963, *p* < 0.0001); - CM-RP *vs* CM-RN (AUC = 0.725, 95% CI 0.624-0.826, *p* < 0.0001); - RP survivors *vs* RP non-survivors (AUC = 0.860, 95% CI 0.773-0.930, *p* < 0.0001). | - | [33]& |
|  |  |  | Malaria severity | Italy | - A cut-off PC value of 50000 [Se = 44 %, Sp = 81 %, PPV = 50 % and NPV = 77 %], 100000 [Se = 89 %, Sp = 57 %, PPV = 47 % and NPV = 92 %], and 150000 [Se = 100 %, Sp = 38 %, PPV = 41 % and NPV = 100 %]. | - | [14] |
|  |  |  | CM | Germany | - CM *vs* UM: AUC = 0.777, 95% CI 0.646 – 0.908, *p* = 0.002 | - | [15] |
|  |  |  | Malaria severity | India | - UM vs SM: Platelet < 150,000 had Se = 47%, Sp = 98.5%, PPV = 96.91%, NPV = 65.02%, PLR = 31.33, NLR = 0.54, Accuracy = 72.75%. | - | [41] |
|  |  |  | Malaria severity | Senegal | - PC < 100000: SM (Se = 85%, Sp = 53%, PPV = 20%, NPV = 96%), CM (Se = 86%, Sp = 61%, PPV = 46%, NPV = 92%), RD (Se = 84%, Sp = 50%, PPV = 31%, NPV = 92%), and SMA (Se = 60%, Sp = 70%, PPV = 21%, NPV = 93%). | Hematological analyzer | [42] |

AM: Asymptomatic malaria, Ang: Angiopoietin, AUC: Area under the curve, CI: Confidence interval, cfDNA: Cell-free DNA, CHI3L1: Chitinase-3-like 1, CM: Cerebral malaria, CMNS: Cerebral malaria non survivors, CKD-EPI eGFR: chronic kidney disease-epidemiology estimated glomerular filtration rate, CK-MB: Cardiac disease creatine kinase muscle-brain type, CLI: Circulatory complement‑lysis inhibitor, CM: Cerebral malaria, CMS: Cerebral malaria survivors, CRP: C-reactive protein, ELISA: Enzyme linked immunosorbent assay, HC: Health control, HMGB1: High mobility group box 1, Hpx: Hemopexin, Hpx: Hemopexin, HRP2: Histidine-rich protein 2, ICAM-1: Intercellular adhesion molecules, IFN: Interferon, IL: Interleukin, IP-10: 10 kDa INF gamma-induced protein, LODS: the Lambaréné Organ Dysfunction Score, Lpc-2: Lipocalin 2, MM: Mild malaria, n.a.: Not applicable, NLR: Negative likelihood ratio, NPV: Negative predictive value, n.s.: Not significant, PCE: Pigment-containing eosinophil, PCG: Pigment-containing granulocyte, PCN: Pigment-containing neutrophil, PCM: Pigment-containing monocyte, PCT: Procalcitonin, PDW: Platelet distribution width, *Pf*: *Plasmodium falciparum*, PLR: Positive likelihood ratio, PPV: Positive predictive value, qPCR: Quantitative Polymerase chain reaction, RBCD: Red blood cell distribution, RBP4: retinol binding protein 4, RD: Respiratory distress, RNA: Ribonucleic acid, RP+: Retinopathy positive, RP-: Retinopathy negative, Se: Sensitivity, sFlt-1: FMS-like tyrosine kinase-1, Sp: Specificity, SM: Severe malaria, SMA: Severe malarial anaemia, TGF: Transforming growth factor, TNF: Tumour necrosis factor; UM: Uncomplicated malaria, vWF: von Willebrand factor

^¶^This study is a meta-analysis

^&^HRP2 and platelet count were used as comparator for evaluate the clinical utility of cfDNA

^#^Biomarkers were evaluated in European travellers diagnosed with severe *Pf* malaria

*Clinical performances of the *Pf* DNA levels were compared to PfHRP2 levels

**References**

[1] Huang H, Ideh RC, Gitau E, et al. Discovery and validation of biomarkers to guide clinical management of pneumonia in african children. *Clin Infect Dis* 2014; 58: 1707–1715.

[2] Ray S, Kumar V, Bhave A, et al. Proteomic analysis of *Plasmodium falciparum* induced alterations in humans from different endemic regions of India to decipher malaria pathogenesis and identify surrogate markers of severity. *J Proteomics* 2015; 127: 103–113.

[3] Abah SE, Burté F, Howell SA, et al. Depleted circulatory complement-lysis inhibitor (CLI) in childhood cerebral malaria returns to normal with convalescence. *Malar J* 2020; 19: 167.

[4] Phu NH, Day N, Diep PT, et al. Intraleucocytic malaria pigment and prognosis in severe malaria. *Trans R Soc Trop Med Hyg* 1995; 89: 200–204.

[5] Kremsner PG, Valim C, Missinou MA, et al. Prognostic value of circulating pigmented cells in African children with malaria. *J Infect Dis* 2009; 199: 142–150.

[6] Salih MM, Eltahir HG, Abdallah TM, et al. Haematological parameters, haemozoin-containing leukocytes in Sudanese children with severe *Plasmodium falciparum* malaria. *J Infect Dev Ctries* 2018; 12: 273–278.

[7] Bhandari S, Krishna S, Patel PP, et al. Diversity and expression of *Plasmodium falciparum var* gene in severe and mild malaria cases from Central India. *Int J Infect Dis* 2021; 103: 552–559.

[8] Duffy F, Bernabeu M, Prasad BH, et al. Meta-analysis of *Plasmodium falciparum* *var* signatures contributing to severe malaria in African children and Indian adults. *mBio* 2019; 10: e00217-19.

[9] Erdman LK, Dhabangi A, Musoke C, et al. Combinations of host biomarkers predict mortality among Ugandan children with severe malaria: A retrospective case-control study. *PLoS ONE* 2011; 6: e17440.

[10] Van Wolfswinkel ME, Hesselink DA, Hoorn EJ, et al. Copeptin does not accurately predict disease severity in imported malaria. *Malar J* 2012; 11: 6.

[11] Van Wolfswinkel ME, De Mendonça Melo M, Vliegenthart-Jongbloed K, et al. The prognostic value of schizontaemia in imported *Plasmodium falciparum* malaria. *Malar J* 2012; 11: 301.

[12] Bhardwaj N, Ahmed M, Sharma S, et al. C-reactive protein as a prognostic marker of *Plasmodium falciparum* malaria severity. *J Vector Borne Dis* 2019; 56: 122–126.

[13] Mohapatra MK, Thomas GA, Kumar Bariha P, et al. Serum procalcitonin: as a Triage tool for severe *Plasmodium falciparum* malaria. *J Trop Dis* 2013; 1: 4.

[14] Righi E, Merelli M, Arzese A, et al. Determination of PCT on admission is a useful tool for the assessment of disease severity in travelers with imported *Plasmodium falciparum* malaria. *Acta Parasitol* 2016; 61: 412–418.

[15] Stauga S, Hahn A, Brattig NW, et al. Clinical relevance of different biomarkers in imported Plasmodium falciparum malaria in adults: A case control study. *Malar J* 2013; 12: 246.

[16] Bhardwaj N, Ahmed MZ, Sharma S, et al. Clinicopathological study of potential biomarkers of *Plasmodium falciparum* malaria severity and complications. *Infect Genet Evol* 2020; 77: 104046.

[17] Chiwakata C, Manegold C, Bönicke L, et al. Procalcitonin as a parameter of disease severity and risk of mortality in patients with *Plasmodium falciparum* malaria. *J Infect Dis* 2001; 183: 1161–1164.

[18] Hesselink DA, Burgerhart JS, Bosmans-Timmerarends H, et al. Procalcitonin as a biomarker for severe *Plasmodium falciparum* disease: A critical appraisal of a semi-quantitative point-of-care test in a cohort of travellers with imported malaria. *Malar J* 2009; 8: 206.

[19] te Witt R, Van Wolfswinkel ME, Petit PL, et al. Neopterin and procalcitonin are suitable biomarkers for exclusion of severe *Plasmodium falciparum* disease at the initial clinical assessment of travellers with imported malaria. *Malar J* 2010; 9: 255.

[20] Conroy AL, Lafferty EI, Lovegrove FE, et al. Whole blood angiopoietin-1 and-2 levels discriminate cerebral and severe (non-cerebral) malaria from uncomplicated malaria. *Malar J* 2009; 8: 295.

[21] Oluboyo A, Chukwu S, Oluboyo B, et al. Evaluation of angiopoietins 1 and 2 in malaria-infested children. *J Environ Public Health* 2020; 2020: 2169763.

[22] Conroy AL, Glover SJ, Hawkes M, et al. Angiopoietin-2 levels are associated with retinopathy and predict mortality in Malawian children with cerebral malaria: A retrospective case-control study. *Cri Care Med* 2012; 40: 952–959.

[23] Lovegrove FE, Tangpukdee N, Opoka RO, et al. Serum angiopoietin-1 and -2 levels discriminate cerebral malaria from uncomplicated malaria and predict clinical outcome in African children. *PLoS ONE* 2009; 4: e4912.

[24] Conroy AL, Hawkes M, McDonald CR, et al. Host biomarkers are associated with response to therapy and long-term mortality in pediatric severe malaria. *Open Forum Infect Dis* 2016; 3: ofw134.

[25] Conroy AL, Phiri H, Hawkes M, et al. Endothelium-based biomarkers are associated with cerebral malaria in Malawian children: A retrospective case-control study. *PLoS ONE* 2010; 5: e15291.

[26] Park GS, Ireland KF, Opoka RO, et al. Evidence of endothelial activation in asymptomatic *Plasmodium falciparum* parasitemia and effect of blood group on levels of von Willebrand factor in Malaria. *J Pediatric Infect Dis Soc* 2012; 1: 16–25.

[27] Erdman LK, Petes C, Lu Z, et al. Chitinase 3-like 1 is induced by *Plasmodium falciparum* malaria and predicts outcome of cerebral malaria and severe malarial anaemia in a case-control study of African children. *Malar J* 2014; 13: 279.

[28] Higgins SJ, Xing K, Kim H, et al. Systemic release of high mobility group box 1 (HMGB1) protein is associated with severe and fatal *Plasmodium falciparum* malaria. *Malar J* 2013; 12: 105.

[29] Seydel KB, Fox LL, Glover SJ, et al. Plasma concentrations of parasite histidine-rich protein 2 distinguish between retinopathy-positive and retinopathy-negative cerebral malaria in Malawian children. *J Infect Dis* 2012; 206: 309–318.

[30] Fox LL, Taylor TE, Pensulo P, et al. Histidine-rich protein 2 plasma levels predict progression to cerebral malaria in malawian children with *Plasmodium falciparum* infection. *J Infect Dis* 2013; 208: 500–503.

[31] Kariuki SM, Gitau E, Gwer S, et al. Value of *Plasmodium falciparum* histidine-rich protein 2 level and malaria retinopathy in distinguishing cerebral malaria from other acute encephalopathies in Kenyan children. *J Infect Dis* 2014; 209: 600–609.

[32] Park GS, Opoka RO, Shabani E, et al. *Plasmodium falciparum* histidine-rich protein-2 plasma concentrations are higher in retinopathy-negative cerebral malaria than in severe malarial anemia. *Open Forum Infect Dis* 2017; 4: ofx151.

[33] Vera IM, Kessler A, Ting LM, et al. Plasma cell-free DNA predicts pediatric cerebral malaria severity. *JCI Insight* 2020; 5: e136279.

[34] Surowiec I, Gouveia-Figueira S, Orikiiriza J, et al. The oxylipin and endocannabidome responses in acute phase *Plasmodium falciparum* malaria in children. *Malar J* 2017; 16: 358.

[35] Tahar R, Albergaria C, Zeghidour N, et al. Plasma levels of eight different mediators and their potential as biomarkers of various clinical malaria conditions in African children. *Malar J* 2016; 15: 337.

[36] Van Wolfswinkel ME, Hesselink DA, Zietse R, et al. Hyponatraemia in imported malaria is common and associated with disease severity. *Malar J* 2010; 9: 140.

[37] Wilson NO, Jain V, Roberts CE, et al. CXCL4 and CXCL10 predict risk of fatal cerebral malaria. *Dis Markers* 2011; 30: 39–49.

[38] Yeo TW, Lampah DA, Kenangalem E, et al. Increased carboxyhemoglobin in adult falciparum malaria is associated with disease severity and mortality. *J Infect Dis* 2013; 208: 813–817.

[39] Mahanta A, Kar SK, Kakati S, et al. Heightened inflammation in severe malaria is associated with decreased IL-10 expression levels and neutrophils. *Innate Immun* 2015; 21: 546–552.

[40] Imwong M, Woodrow CJ, Hendriksen ICE, et al. Plasma concentration of parasite DNA as a measure of disease severity in falciparum malaria. *J Infect Dis* 2015; 211: 1128–1133.

[41] Dhangadamajhi G, Panigrahi S, Roy S, et al. Effect of *Plasmodium falciparum* infection on blood parameters and their association with clinical severity in adults of Odisha, India. *Acta Trop* 2019; 190: 1–8.

[42] Gérardin P, Rogier C, Ka AS, et al. Prognostic value of thrombocytopenia in African children with falciparum malaria. *Am J Trop Med Hyg* 2002; 66: 686–691.
