# Supplementary material for: The spectrum of clinical biomarkers in severe malaria and new avenues for exploration
Source: Virulence. 2022 Aug 29;13(1):634–53. doi: 10.1080/21505594.2022.2056966 (PMC9427047; doi:10.1080/21505594.2022.2056966)
Supplement: Supplemental Material [file KVIR_A_2056966_SM1249.zip › supplementary/Clean_ Supplemental material 3.docx]

**Supplemental material 3**. Performances of the proposed biomarkers for severe *Pv* and *Pk* malaria

**Table S3**. (references are presented at the end of this supplemental material)

| **Biomarker** | **Origin** | **Nature** | **Endpoint** | **Country** | **Major findings from the study** | **Measurement (body fluid)** | **Ref.** |
| --- | --- | --- | --- | --- | --- | --- | --- |
| ***Plasmodium vivax*** | | | | | | | |
| Superoxide Dismutase-1 | Host | Protein | Malaria severity | Brazil | - Levels of SOD-1 was increased with malaria severity; - MM *vs* SM (AUC = 0.980, *p* < 0.001, Se = 100 %, Sp = 82.5 %); - AM *vs* MM (AUC = 0.966, *p* < 0.001, Se = 100 %, Sp = 80 %). | ELISA (Plasma) | [1] |
|  |  |  | Malaria severity | India | - 31 proteins were differentially expressed between SM and UM; - HC *vs* UM (AUC = 0.6094, 95% CI 0.4200 - 0.7988); - HC *vs* SM (AUC = 0.8844, 95% CI 0.7688 - 0.9999); - SM *vs* UM (AUC = 0.9088, 95% CI 0.8172 – 1.0000). | ELISA (Plasma) | [2]* |
| Apoliprotein E | Host | Protein | Malaria severity | India | - 31 proteins were differentially expressed between SM and UM; - HC *vs* UM (AUC = 0.747, 95% CI 0.675 - 0.818); - HC *vs* SM (AUC = 0.811, 95% CI 0.683 - 0.938); - SM *vs* UM (AUC = 0.656, 95% CI 0.521 - 0.791). | ELISA (Plasma) | [2]* |
| Haptoglobin | Host | Protein | Malaria severity | India | - 31 proteins were differentially expressed between SM and UM; - HC *vs* UM (AUC = 0.818, 95% CI 0.760 - 0.876); - HC *vs* SM (AUC = 0.830, 95% CI 0.740 - 0.919); - SM *vs* UM (AUC = 0.688, 95% CI 0.577 - 0.800). | ELISA (Plasma) | [2]* |
| Serum amyloid A | Host | Protein | Malaria severity | India | - 31 proteins were differentially expressed between SM and UM; - HC *vs* UM (AUC = 0.931, 95% CI 0.898 - 0.963); - HC *vs* SM (AUC = 0.959, 95% CI 0.931 - 0.986); - SM *vs* UM (AUC = 0.609, 95% CI 0.505 - 0.714). | ELISA (Plasma) | [2]* |
| Titin | Host | Protein | Malaria severity | India | - 31 proteins were differentially expressed between SM and UM; - HC *vs* UM (AUC = 0.625, 95% CI 0.3115 - 0.9385); - HC *vs* SM (AUC = 0.875, 95% CI 0.7091 - 1); - SM *vs* UM (AUC = 0.75, 95% CI 0.5168 - 0.9832). | ELISA (Plasma) | [2]* |
| Vitronectin | Host | Protein | Malaria severity | India | - 31 proteins were differentially expressed between SM and UM; - HC *vs* UM (AUC = 0.65, 95% CI 0.4102 - 0.8898); - HC *vs* SM (AUC = 0.825, 95% CI 0.6442 - 1); - SM *vs* UM (AUC = 0.76, 95% CI 0.5391 - 0.9809). | ELISA (Plasma) | [2]* |
| Tumour necrosis factor-alpha (TNF- α) | Host | Protein | Malaria severity | Brazil | - Levels of TNF-α was increased with malaria severity; - MM *vs* SM (AUC = 0.887, *p* < 0.001, Se = 70.6 %, Sp = 92 %). | ELISA (Plasma) | [1] |
|  |  |  | CM | Pakistan | - TNF-α was significantly higher in CM compared to UM; - CM *vs* UM (AUC = 0.89, 95% CI 0.80 - 0.94, *p* < 0.0001; Cut-off point > 91.40 pg/mL with a Se = 98.8 % and Sp = 82.0 %). | ELISA (Plasma) | [3] |
| Protein carbonylation (PC) | Host | Biological process | Malaria severity | India | - PC was significantly higher in SM compared to UM and FC; - UM *vs* SM (AUC = 0.86, *p* = 0.001, Cut-off = 8.55 nM/mg with a Se = 68.7 % and Sp = 88.6 %). | Di-nitrophenyl hydrazine assay | [4] |
| Lipid peroxidation (LPO) | Host | Biological process | Malaria severity | India | - LPO was significantly higher in SM compared to UM and FC; - UM *vs* SM (AUC = 0.80, *p* = 0.001, Cut-off = 2.41 nM with a Se = 87.5 % and Sp = 67.0 %). | Thiobarbituric acid reaction assay | [4] |
| Platelet count | Host | Cellular | Malaria severity | India | - Platelet count was significantly lower in SM compared to UM and FC patients; - UM *vs* SM (AUC = 0.65, *p* = 0.07, Cut-off = 112000 with a Se = 62.5 % and Sp = 68.7 %). | ELISA (Plasma) | [4] |
| C-reactive protein | Host | Protein | Malaria severity | India | - CRP was significantly higher in UM and SM compared to FC, but no difference between UM and SM (*p* = 0.259); - UM *vs* SM (AUC = 0.74, *p* = 0.001, Cut-off = 42.5 ng/mL with a Se = 68.7 % and Sp = 81.0 %). | ELISA (Plasma) | [4] |
| Anti-oxidant status | Host | Biological process | Malaria severity | India | - LPO was significantly lower in SM compared to UM and FC; - UM *vs* SM (AUC = 0.91, *p* = 0.001, Cut-off = 332.5 µmol/L with a Se = 93.7 % and Sp = 78.5 %) | FRAP assay | [4] |
| Cells adhesion molecules (CAMs) | Host | Proteins | CM | Pakistan | - sICAM-1, sVCAM-1 were significantly higher in CM compared to UM, but no difference for E-selectin; - CM vs UM (For ICAM-1: AUC = 0.63, 95% CI 0.55 - 0.71, *p* < 0.004; Cut-off > 694.40 with a Se = 91.5% and Sp = 75.0%; For VCAM-1: AUC = 0.84, 95% CI 0.78 - 0.90, *p* < 0.0001; Cut-off > 930.5 with a Se = 99.0 % and Sp = 80.0 %) | ELISA (Plasma) | [3] |
| Interleukin 10 | Host | Protein | CM | Pakistan | - IL-10 was significantly higher in CM compared to UM; - CM *vs* UM (AUC = 0.79, 95% CI 0.73 - 0.86, *p* < 0.0001; Cut-off > 694.4 with a Se = 91.5 % and Sp = 75.0 %) | ELISA (Plasma) | [3] |
| Interleukin 6 | Host | Protein | CM | Pakistan | - IL-6 was significantly lower in CM compared to UM; - CM *vs* UM (AUC = 0.50, 95% CI 0.41 - 0.60, *p* = 0.94; Cut-off < 47534 with a Se = 50.0 % and Sp = 51.0 %) | ELISA (Plasma) | [3] |
| Angiopoietins 1/2 | Host | Protein | Malaria severity | Brazil | - Ang-1 was significantly lower in SM compared to HC (*p* = 0.005); - Ang-2 and Ang-2/Ang-1 ratio were significantly higher in SM compared to HC (*p* = 0.032 and *p* = 0.002, respectively); - SM *vs* HC (All patients - Ang-2: AUC = 0.667, Cut-off = 5.9 ng/mL with a Se = 77.8 % and Sp = 50.0 %; All patients - Ang-2/Ang-1 ratio: AUC = 0.737, Cut-off = 0.75 with Se = 61.1 % and Sp = 83.9 %; Patients with platelets <75,000/µL- Ang-2: AUC = 0.833, Cut-off = 8.4 ng/mL with a Se = 87.5% and Sp = 66.7 %; Patients with platelets <75,000/µL - Ang-2/Ang-1 ratio: AUC = 0.881, Cut-off = 1.20 with a Se = 75.0 % and Sp = 90.5 %; Patients with platelets >75,000/µL- Ang-2: AUC = 0.527, Cut-off = 3.85 ng/mL with a Se = 80.0 % and Sp = 29.3 %; Patients with platelets >75,000/µL - Ang-2/Ang-1 ratio: AUC = 0.645, Cut-off = 0.30 with a Se = 60.0 % and Sp = 68.3 %). | ELISA (Serum) | [5]‡ |
|  |  |  | CM | India | - CMNS *vs* MM (Ang-1: AUC = 0.3472, Ang-2: AUC = 0.9462, Ang-2/Ang-1 ratio = 0.8996); - CMNS *vs* CMS (Ang-1: AUC = 0.5003, Ang-2: AUC = 0.7564, Ang-2/Ang-1 ratio = 0.6853). | ELISA (Plasma) | [6] |
| Platelet count | Host | Cell | Malaria severity | India | - A platelet count values of < 150 × 10^3^ (Se = 79.8%, Sp = 39.8%, PPV = 72.3%, NPV = 50%), 50-149 × 10^3^ (Se = 62.5%, Sp = 51%, PPV = 63.4%, NPV = 50%), 20-49 × 10^3^ (Se = 56%, Sp = 72.9%, PPV = 77.4%, NPV = 50%), and < 20 × 10^3^ (Se = 50%, Sp = 85%, PPV = 85%, NPV = 50%). | Haematological analyser | [7] |
| ***Plasmodium knowlesi*** | | | | | | | |
| Pigment-containing neutrophil (PCN) | Host | Cell | Malaria severity | Malaysia | - PCN was significantly higher in SM compared to UM (*p* < 0.0001); - AUC = 0.8561 (95% CI 0.7328-0.9794), but no difference with parasite count (AUC = 0.8261 and 0.8476) and platelet count (AUC = 0.7723). | Light microscopy | [8] |
| Platelet count | Host | Cell | Malaria severity | Malaysia | - Platelet count was significantly lower in SM *vs* UM (*p* = 0.0004); - Thrombocytopenia (45,000/µL) was strongly and independently associated with SM (OR = 5.27, *p* = 0.0004); - AUC = 0.7723 (95% CI 0.6775-0.8671), but no difference with parasite count (AUC = 0.8261 and 0.8476) and PCN (AUC = 0.8561). | Haematological analyser | [8] |

AM: Asymptomatic malaria, Ang: Angiopoietin, AUC: Area under the curve, CI: Confidence interval, CM: Cerebral malaria, CMNS: Cerebral malaria non survivors, CMS: Cerebral malaria survivors, CRP: C reactive protein, ELISA: Enzyme-linked immunosorbent assay, FC: Febrile Control, HC: Healthy control, ICAM-1: Intercellular adhesion molecule-1, IL: Interleukin, LPO: Lipid peroxidation, MM: Mild malaria, NPV: Negative predictive value, Se: Sensitivity, PC: Protein carbonylation, PCN: Pigment-containing neutrophils, *Pk*: *Plasmodium knowlesi*, *Pv*: *Plasmodium vivax*, PPV: Positive predictive value, SM: Severe malaria, SOD-1: Superoxide Dismutase-1, Sp: Specificity, TNF-α: Tumour necrosis factor alpha, UM: Uncomplicated malaria, VCAM-1: Vascular adhesion molecule-1

*The biomarkers were quantified using proteomic analysis (surface plasmon resonance-based quantification, coupled with ELISA

‡In this study, the authors determined the patterns of Ang-2, and Ang-2/Ang-1 ratio according to the level of platelet count

**References**

[1] Andrade BB, Reis-Filho A, Souza-Neto SM, et al. Plasma superoxide dismutase-1 as a surrogate marker of vivax malaria severity. *PLoS Med* 2010; 4: e650.

[2] Ray S, Patel SK, Venkatesh A, et al. Clinicopathological analysis and multipronged quantitative proteomics reveal oxidative stress and cytoskeletal proteins as possible markers for severe vivax malaria. *Sci Rep* 2016; 6: 24557.

[3] Raza A, Ghanchi NK, Zubairi ABS, et al. Tumor necrosis factor -α, interleukin-10, intercellular and vascular adhesion molecules are possible biomarkers of disease severity in complicated *Plasmodium vivax* isolates from Pakistan. *PLoS ONE* 2013; 8: e81363.

[4] Aqeel S, Naheda A, Raza A, et al. Biomarkers of disease severity in vivax malaria. *Parasitol Res* 2021; 120: 1437–1446.

[5] Gomes LT, Alves- ER, Rodrigues-Jesus C, et al. Angiopoietin-2 and angiopoietin-2/angiopoietin-1 ratio as indicators of potential severity of *Plasmodium vivax* malaria in patients with thrombocytopenia. *PLoS ONE* 2014; 9: e109246.

[6] Jain V, Lucchi NW, Wilson NO, et al. Plasma levels of angiopoietin-1 and -2 predict cerebral malaria outcome in Central India. *Malar J* 2011; 10: 383.

[7] Tanwar GS, Khatri PC, Chahar CK, et al. Thrombocytopenia in childhood malaria with special reference to *P. vivax* monoinfection: A study from Bikaner (Northwestern India). *Platelets* 2012; 23: 211–216.

[8] Willmann M, Ahmed A, Siner A, et al. Laboratory markers of disease severity in *Plasmodium knowlesi* infection: A case control study. *Malar J* 2012; 11: 363.
